# Protein profiling identified key chemokines that regulate the maintenance of human pluripotent stem cells

**DOI:** 10.1038/s41598-017-15081-6

**Published:** 2017-11-06

**Authors:** Zongmin Jiang, Yonggang Li, Xinglai Ji, Yiyuli Tang, Haijing Yu, Lei Ding, Min Yu, Qinghua Cui, Ming Zhang, Yanping Ma, Meizhang Li

**Affiliations:** 1 Laboratory of Biochemistry and Molecular Biology, School of Life Sciences, Yunnan University, Kunming, Yunnan 650091 China; 2 Key Laboratory of Molecular Cancer Biology, Yunnan Education Department, Kunming, Yunnan 650091 China; 3 Department of Reproduction and Genetics, the First People’s Hospital of Yunnan Province, Kunming, Yunnan 650032 China; 4 State Key Laboratory for Conservation and Utilization of Bio-Resources in Yunnan, Yunnan University, Kunming, Yunnan 650091 China; 5 Yunnan Key Laboratory of Stem Cell and Regenerative Medicine, Institute of Molecular and Clinical Medicine, Kunming Medical University, Kunming, Yunnan 650500 China

## Abstract

Microenvironment (or niche)-providing chemokines regulate many important biological functions of tissue-specific stem cells. However, to what extent chemokines influence human pluripotent stem cells (hPSCs) is not yet completely understood. In this study, we applied protein array to screen chemokines found within the cytokine pool in the culture supernatant of hPSCs. Our results showed that chemokines were the predominant supernatant components, and came from three sources: hPSCs, feeder cells, and culture media. Chemotaxis analysis of IL-8, SDF-1α, and IP-10 suggested that chemokines function as uniform chemoattractants to mediate *in vitro* migration of the hPSCs. Chemokines mediate both differentiated and undifferentiated states of hPSCs. However, balanced chemokine signaling tends to enhance their stemness *in vitro*. These results indicate that chemokines secreted from both stem cells and feeder cells are essential to mobilize hPSCs and maintain their stemness.

## Introduction

Human pluripotent stem cells (hPSCs) are unique cell types that maintain self-renewal and pluripotency during early embryonic development^1–3^. Embryonic stem cells (ESCs) and induced pluripotent stem cells (iPSCs) are two known PSCs: the former are derived from the inner cell mass of early blastocysts, whereas the latter are dedifferentiated from mature somatic cells through genetic reprogramming^2,4^. Both ESCs and iPSCs have demonstrated great potential in regenerative medicine due to their unlimited capability to be differentiated into almost all types of tissues^5,6^. Specialized microenvironments (niches) are suggested to provide the multiple extracellular signals necessary to maintain the stemness of hPSCs^7–10^. Previous studies have indicated that niche-dependent extracellular matrices (ECMs) facilitate the self-renewal and pluripotency of PSCs^11,12^, with growth factors (such as basic fibroblast growth factor, bFGF, and epidermal growth factor, EGF) regulating their growth and survival^13^. Recent studies also suggest that niche-providing cytokines are possibly involved in the maintenance of PSCs^14^.

Chemokines are small secreted cytokines responsible for leukocyte trafficking during host defense immune response^15,16^. They are classified into CXC, CC, C, and CX3C subfamilies based on the conserved cysteine motif at the N-terminus of their polypeptide sequences^17^. Chemokine receptors are generally seven-transmembrane G protein-coupled receptors (GPCRs)^18^. Binding of chemokines to their receptors induces the G protein-mediated downstream signaling pathways and regulates cellular survival, proliferation, and migration^19–22^. Chemokines are key regulators of tissue-specific stem cells^23,24^. As the first identified niche-dependent chemokine, CXC chemokine SDF-1α (or CXCL12) regulates the homing and migration of hematopoietic progenitor cells (HPCs) in bone marrow^25,26^. CXCL12 receptor CXCR4 is expressed in HPCs^27^, with gene knockout of CXCR4 or blockage of CXCR4-specific antagonist AMD3100 able to inhibit the homing and migration of HPCs^28,29^. Our previous study found that the CXCL12-CXCR4 axis regulates the proliferation, migration, and survival of neural stem cells^30^. Similar roles of CXCL12-CXCR4 have been reported for many other multi-potential stem cells, such as primordial germ cells, endothelial progenitor cells, epithelial progenitor cells, neural stem cells, mesenchymal stem cells, liver oval stem cells, and cancer stem cells^31–38^. In addition, many other chemokines influence the biology of tissue-specific stem cells^39^. For example, CC chemokine MIP-1α (CCL3) inhibits the proliferation and mobilization of HPCs^40,41^. CXC chemokines IL-8 (CXCL8) and GRO (CXCL2) can enhance the mobilization of HPCs and human CNS stem cells^42–46^. In degenerative diseases, upregulated CC chemokines MCP-1 (CCL2), MIP-1α (CCL3), and RANTES (CCL5) are possibly involved in the migration of endothelial progenitor cells during tissue repair^47–49^, with MCP-1 and MIP-1α able to mobilize mesenchymal stem cells into ischemic brain lesions^50,51^. The above studies suggest that chemokines are niche-dependent signals that help maintain tissue-specific stem cells.

Recent research also suggests that chemokines maintain the stemness of hPSCs^52,53^. It has been reported that GROα potentially regulates the self-renewal of human ESCs in *in vitro* culture^54^. Furthermore, MCP-1 appears to cooperate with leukemia inhibitory factor to promote the stemness of mouse iPSCs through mediation of the Stat3-pathway^55^. SDF-1α has been reported to induce the migration of mouse ESCs and enhances their survival^56^. Human MCP-1 increases the expression of pluripotent marker genes through the phosphorylation of the signal transducer and activator of transcription 3 in iPSCs^57^. IL-8 and/or GROa also support the maintenance and proliferation of hPSCs^54,58^. In the current study, we performed high-throughput screening to identify three key chemokines (IL-8, IP-10, and SDF-1α) that regulate the mobilization and stemness of hPSCs.

## Results

### Successful establishment of new hESC cell line

**Figure 1:**
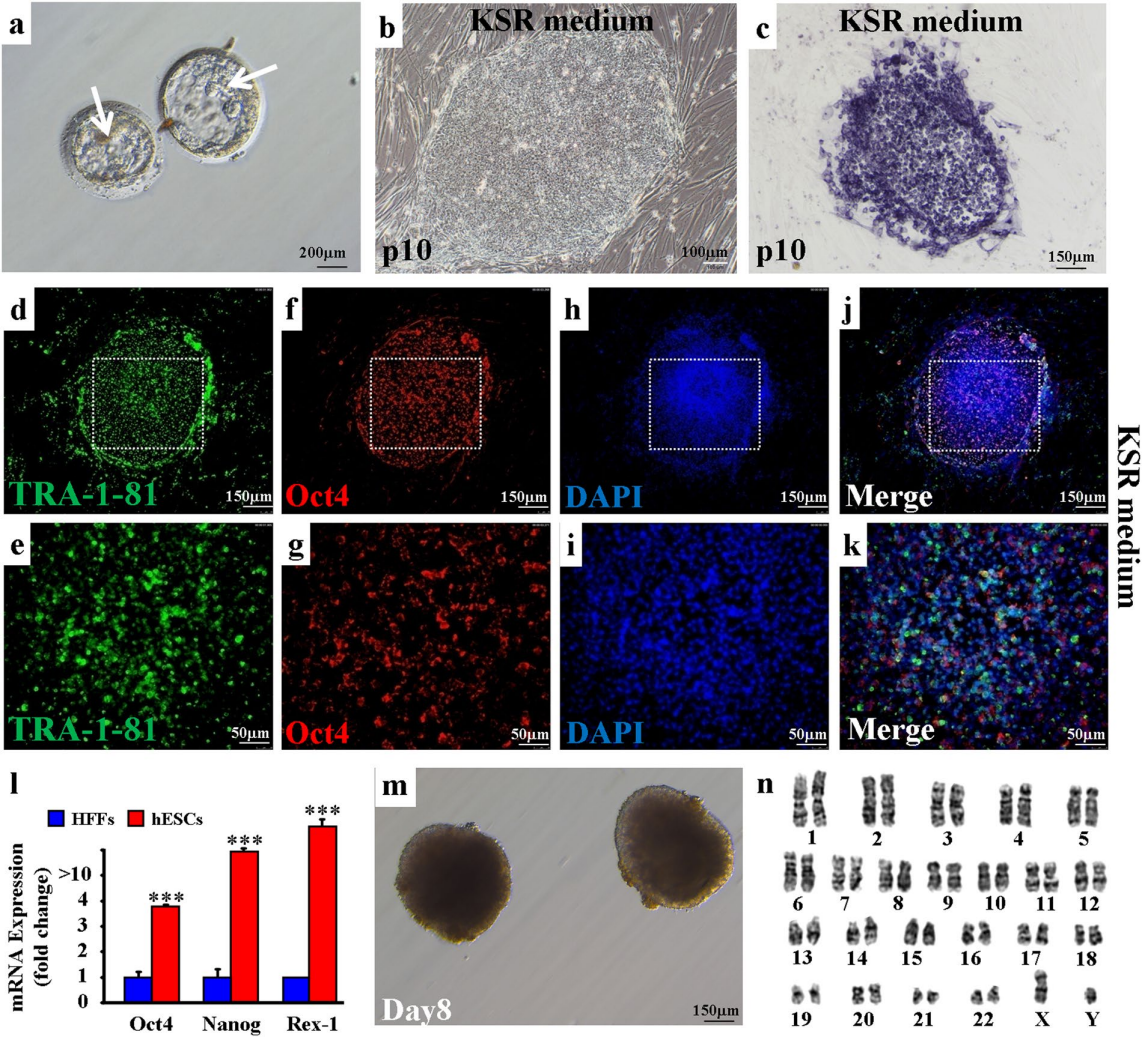
Development and characterization of new hESCs. (**a)** Imaging of two human blastocysts at post-fertilization day 6 *in vitro*. Inner mass cells are indicated by arrows. Scale bar, 200 μm. **(b)** hESC-derived clones were obtained from inner mass cells after a 10-passage (p10) culture in the KSR medium. Scale bar, 100 μm. **(c)** Cells from (**b**) demonstrated positive staining of alkaline phosphatase. Scale bar, 150 μm. **(d**–**k)** Immunofluorescence of pluripotency markers TRA-1-81 and Oct4 expressed in p10 hESCs. TRA-1-81 (**d**–**e**); Oct4 (**f**,**g**); DAPI (**h**,**i**); and Merge (**j**,**k**). Scale bars, 150 μm (**d**,**f**,**h**,**j**) and 50 μm (**e**,**g**,**i**,**k**). **(l)** mRNA levels of pluripotency markers *Oct4, Nanog, and Rex-1* in p10 hESCs. ***p < 0.001, n = 3 individual experiments. Error bars indicate sem. **(m)** hESCs could be differentiated into embryonic bodies (EBs). Scale bar, 150 μm. **(n)** hESCs show normal human male karyotype.

We applied fertilized human eggs to generate new human embryonic stem cells (hESCs). At post-fertilization day 6, the eggs exhibited normal development of the intact inner cell mass (ICM), trophoblast cells, and pellucid zone *in vitro* (Fig. 1a). After pronase digestion and direct dissection, ICM cells were dissociated and cultured in Nunc 4-well plates supplied with KSR culture medium. Mitomycin-treated human foreskin fibroblasts (HFFs) were simultaneously provided as the feeder cells. We found that ICM cells could generate new clones, with cells from clones sustaining the capability to continuously form new clones for multiple generations (Fig. 1b). In addition, clone-derived cells, for example at the 10^th^ passage (p10), demonstrated positive alkaline phosphatase activity (Fig. 1c). These observations indicate that the clone-derived cells were potential hESCs.

**Figure 2:**
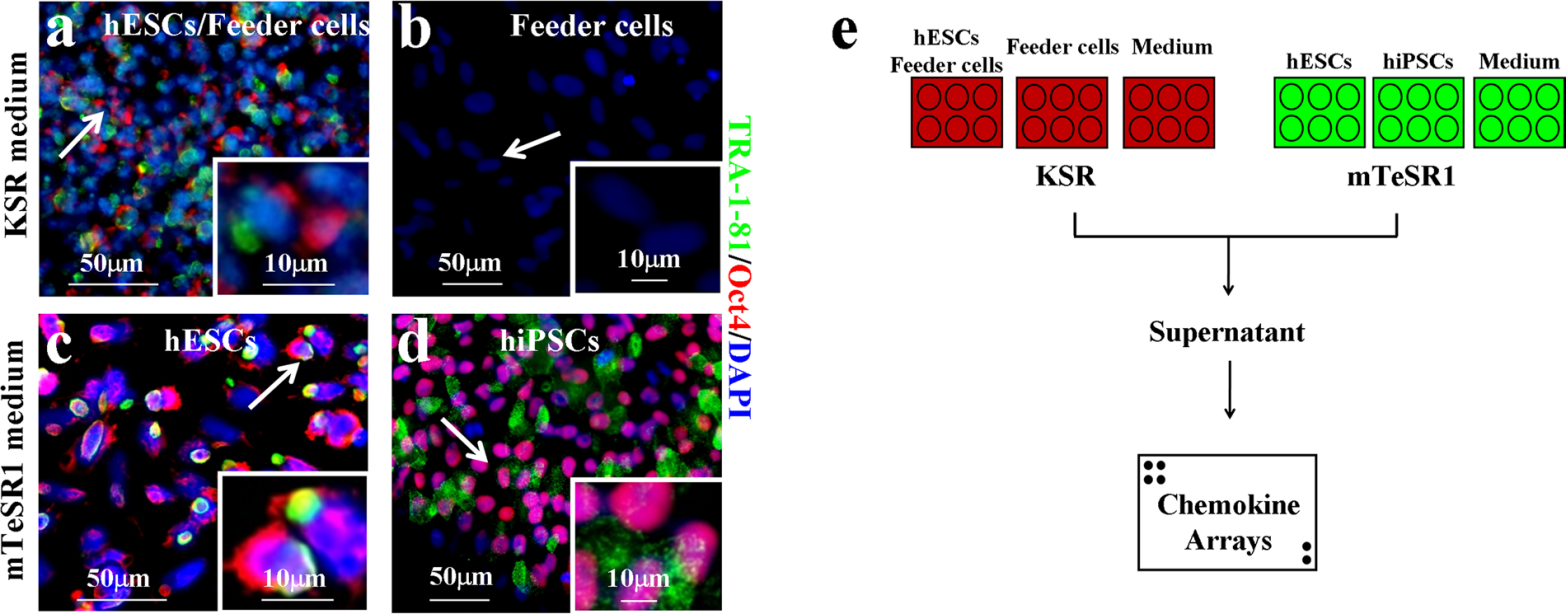
Chemokine screening strategy for hPSCs. (**a**–**d)** Immunofluorescence of pluripotency markers TRA-1-81 and Oct4 on hPSCs. KSR medium (**a**,**b**); mTeSR1 medium (**c**,**d**); hESCs (**a**,**c**); hiPSCs (**d**); Feeder cells (**b**); Scale bars, 50 μm (**a**–**d**; arrows) and 10 μm (insets). Cells were counterstained with DAPI. **(e)** Chemokine screening strategy for hPSCs cultured in KSR (red) or mTeSR1 (green) media.

To verify their stemness, we examined the protein expressions of pluripotency markers Oct4, TRA-1-81, Nanog, and TRA-1-60 in the hESCs, and found them all to be highly expressed (Figs 1d–k, 2a,b and S1). We also observed that the mRNA levels of the pluripotency genes *Oct4*, *Nanog*, and *Rex-1* in the hESCs were higher than those in the HFFs (Fig. 1l). The predominant expression of pluripotency protein markers in the hESCs support that these cells maintained their stemness when cultured in KSR medium. To determine their pluripotency, we also examined whether hESC clumps could be differentiated into three germ layers in the embryoid bodies (EBs) (Fig. 1m). Firstly, the hESCs were transplanted into NOD SCID mouse leg muscles for 48-d differentiation *in vivo*, with the normal three germ layer-tissue differentiations then detected in the formed teratomas by histopathological analyses (Fig. S2), indicating that the established hESC line possessed pluripotency. In addition, quantitative PCR demonstrated higher mRNA expression of the three germ layer markers *AFP*, *GATA4*, *GATA6*, *FOXA2*, *Sox17* (endoderm), *T*, *MIXL1*, *TBX1*, *c-actin* (mesoderm), *Nestin*, *Sox1*, *NeuroD1*, and *PAX6* (ectoderm) in differentiated EBs than in undifferentiated hESCs (Fig. S3a). Significantly higher expressions of the *Oct4* and *Nanog* pluripotency genes were found in the hESCs than in differentiated EBs (Fig. S3b). Consistently, we observed high protein expression of the germ layer markers AFP and GATA4 (endoderm), desmin and actin (mesoderm), and nestin and βIII-tubulin (ectoderm) in the EBs (Fig. S4). These observations suggest that pluripotent hESCs could be differentiated into endoderm, mesoderm, and ectoderm *in vitro*. Finally, the hESCs were found to exhibit a normal male karyotype (Fig. 1n), supporting the successful establishment of a new hESC cell line.

### Maintenance of pluripotent stem cells (PSCs) *in vitro*

We next examined the stemness of the established hESCs cultured in mTeSR1 medium (Fig. S5a). Again, the hESCs demonstrated AP-positive staining, as observed in those cultured in the KSR medium (Fig. S5b). Immunofluorescence staining showed that hESCs were expressed by pluripotency markers Oct4, TRA-1-81, Nanog, and TRA-1-60 (Figs 2c, S5 c–j and S6). To compare established hESCs with other pluripotent stem cells, we cultured known human induced pluripotent stem cell line hNF C11 in mTeSR1 medium (Fig. S5k)^59^, and found that these cells were AP-positive (Fig. S5l) and expressed pluripotency markers Oct4, TRA-1-81, Nanog, and TRA-1-60 (Figs 2d, S5 m–t and S7). These observations further support that hPSCs, such as hESCs and hiPSCs, can be normally cultured under mTeSR1 conditions and sustain their stemness *in vitro*.

### Identification of chemokines secreted from hPSCs and feeder cells

To screen chemokines that potentially regulate hPSC biology, we collected supernatants from the above hESC or hiPSC cultures for chemokine screening (Fig. 2e). In total, 38 chemokines had signals stronger than the background signals of the protein array membrane (Table 1 and Fig. S8). To quantify these signals, relative signal intensity (RSI) was defined by normalizing the signals with background intensities (see Methods and Materials). Under mTeSR1 conditions, 30 chemokines demonstrated obviously higher RSIs in the hESC-mTeSR1 culture compared with those in the mTeSR1 medium only (Fig. S9a). However, both the intensities and spectra of the chemokines secreted from hiPSCs exhibited decreasing expression in the hiPSC-mTeSR1 culture compared with those in the mTeSR1 medium only (Fig. S9b). These results suggest a predominant chemokine component from hPSCs themselves in the culture *in vitro*.

**Table 1:** The relative signal intensities (RSIs) of chemokines.

Number	Symbol	Name	Receptor	KSR medium			mTeSR1 medium			
				hESCs and Feeder cells	Feeder cells	Medium	hESCs	hiPSCs	Medium	Notes
			***CC subfamily***							
1	CCL23	CCL23	CCR1	—	—	122.95	142.46	—	—	G1; G2
2	CCL28	CCL28	CCR3, CCR10	—	—	—	149.71	16.60	—	F1; F2
3	CTACK	CCL27	CCR10	291.48	131	110.97	242.79	102.15	118.93	H1; H2
4	Eotaxin1	CCL11	CCR2, CCR3, CCR5	239.46	122.5	88.66	151.38	34.61	25.39	K1; K2
5	Eotaxin2	CCL24	CCR3	154.11	86	103.12	155.28	32.68	—	L1; L2
6	Eotaxin3	CCL26	CCR3	—	72.5	113.45	—	12.09	47.10	A3; A4
7	MCP1	CCL2	CCR2	535.33	289	130.39	321.38	237.88	29.56	L3; L4
8	MCP2	CCL8	CCR2B	444.29	232.5	—	—	8.23	32.91	A5; A6
9	MCP3	CCL7	CCR2	766.17	309	—	122.40	6.30	—	B5; B6
10	MCP4	CCL13	CCR2, CCR3, CCR5	448.35	185	—	149.71	—	—	C5; C6
11	MDC	CCL22	CCR4	199.63	92.5	—	224.40	15.31	30.40	D5; D6
12	MIP-1α	CCL3	CCR1	276.03	82	87.84	241.12	17.88	38.75	F5; F6
13	MIP-1 β	CCL4	CCR1, CCR5	289.85	183	96.51	241.68	41.04	73.83	G5; G6
14	MIP-1δ	CCL15	CCR1, CCR3	121.60	48.5	—	115.15	—	23.72	H5; H6
15	MIP-3α	CCL20	CCR6	403.65	49.5	—	265.09	10.81	—	I5; I6
16	MIP-3β	CCL19	CCR7	178.50	69.5	125.43	197.09	23.67	—	J5; J6
17	MPIF1	CCL23	CCR1	111.03	61	168.40	184.27	25.60	25.39	K5; K6
18	HCC4	CCL16	CCR1, CCR2, CCR5, CCR8	267.09	132	114.28	394.95	16.60	29.56	F3; F4
19	I-309	CCL1	CCR8	119.97	78	—	160.86	—	—	G3; G4
20	PARC	CCL18	CCR8, PITPNM3, GPR30	—	—	—	—	9.52	33.74	A7.A8
21	RANTES	CCL5	CCR5	121.60	45	—	—	17.88	52.11	B7; B8
22	TARC	CCL17	CCR4	—	—	—	—	—	66.31	E7; E8
23	TECK	CCL25	CCR9	—	—	—	—	8.23	—	F7; F8
			***CXC subfamily***							
24	BLC	CXCL13	CXCR5	—	49.5	—	266.20	15.95	—	E1; E2
25	CXCL16	CXCL16	CXCR6	215.07	90	—	201.55	21.74	—	I1; I2
26	ENA78	CXCL5	CXCR2	359.76	209	—	241.12	201.86	23.72	J1; J2
27	GCP2	CXCL6	CXCR1, CXCR2	740.16	392	102.71	432.85	151.04	62.14	C3; C4
28	GRO	CXCL2	CXCR1, CXCR2	1041.71	427	—	614.56	265.54	32.07	D3; D4
29	GRO-α	CXCL1	CXCR2	820.63	377	—	573.31	51.98	23.72	E3; E4
30	IL-8	CXCL8	CXCR1, CXCR2	773.48	403.5	—	484.13	17.24	—	I3; I4
31	IP-10	CXCL10	CXCR3	585.72	259	135.35	466.85	29.46	56.29	J3; J4
32	I-TAC	CXCL11	CXCR3, CXCR7	255.71	155.5	115.10	401.64	25.60	42.09	H3; H4
33	MIG	CXCL9	CXCR3	164.68	65.5	—	236.66	14.67	41.26	E5; E6
34	SDF-1α	CXCL12	CXCR4	—	—	—	—	—	—	C7; C8
35	SDF-1β	CXCL12	CXCR4	—	—	—	—	11.45	26.22	D7; D8
36	NAP2	CXCL7	—	256.53	79	175.01	239.45	47.47	32.91	L5; L6
			***C subfamily***							
37	XCL1	XCL1	XCR1	341.87	213	107.67	379.90	88.64	60.47	K3; K4
			***CX3 C subfamily***							
38	Fractalkine	CX3CL1	CX3CR1	177.68	115	145.68	200.99	27.53	46.27	B3; B4

**Figure 3:**
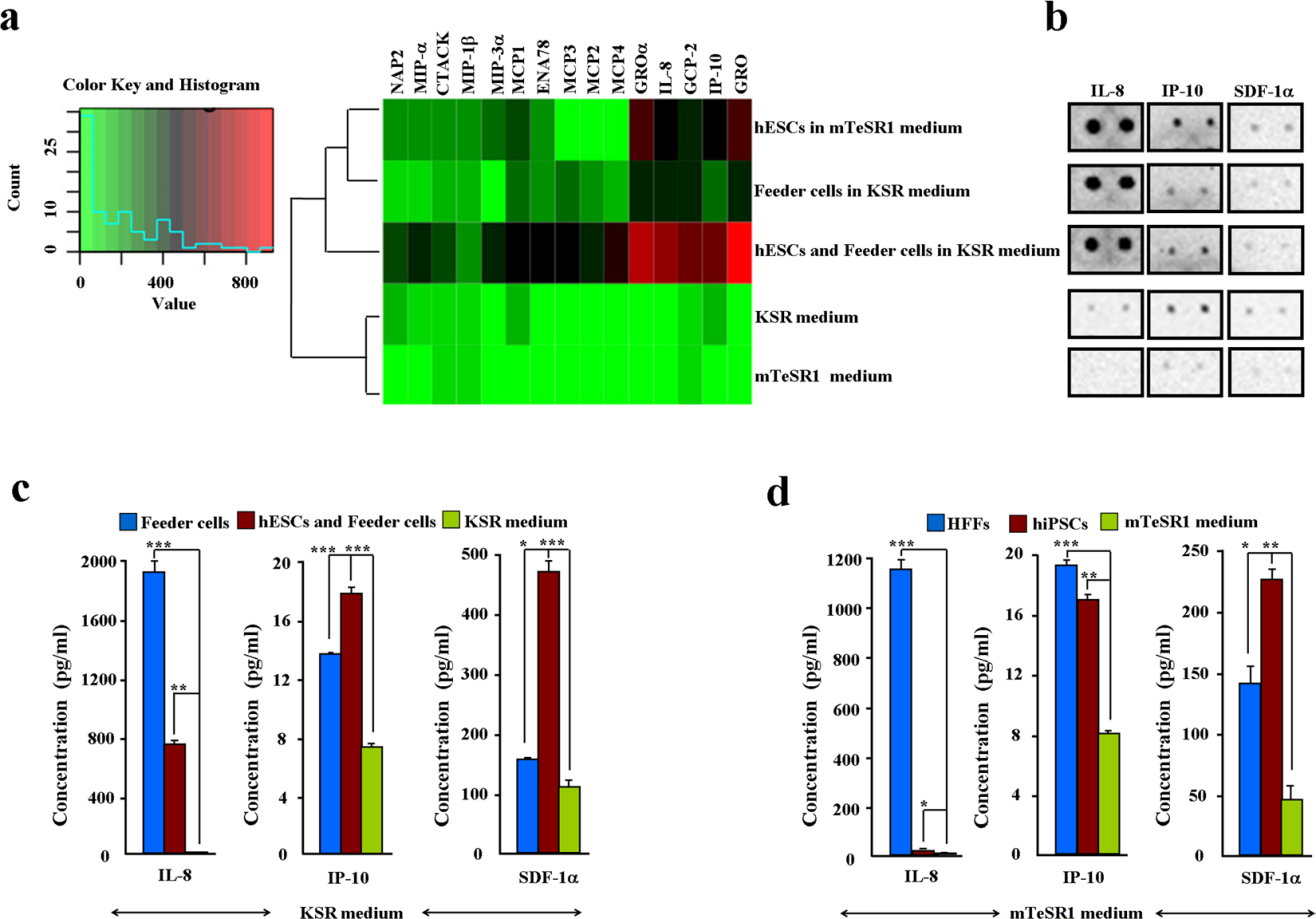
Identification of chemokines secreted from hPSCs and feeder cells. (**a)** Heatmap of 15 identified chemokines. Color key indicates relative signal intensity (RSI) ranges from high (red) to low (green) values. **(b)** Duplicated signals of chemokines IL-8, IP-10, and SDF-1α on protein membranes. **(c**,**d)** Protein concentrations of chemokines IL-8, IP-10, and SDF-1α were examined by ELISA. KSR medium (**c**); mTeSR1 medium (**d**).

We next examined the chemokines secreted from feeder cells. Results showed that feeder cells secreted chemokines under KSR conditions (Fig. S9c). Co-culturing feeder cells with hESCs resulted in the secretion of more diverse and higher concentrations of chemokines than when feeder cells were cultured alone (Fig. S9d). These observations suggest that microenvironments represented by feeder cells significantly contributed to the chemokine components in the hESCs under KSR culture conditions. To verify this hypothesis, we compared the hierarchical clustering-secretion spectra of 15 chemokines between the two different hESC cultures (Fig. 3a). We found that hESCs and feeder cells co-cultured under KSR conditions secreted the maximal concentration of chemokines. Chemokines GRO, GROα, IL-8, GCP-2, and IP-10 were the most highly concentrated (Fig. 3a). These observations were confirmed by detecting representative chemokines IL-8, IP-10, and SDF-1α by ELISA (Fig. 3c,d). All three chemokine proteins were detected in the supernatants (Fig. 3c,d). Interestingly, results showed a relatively higher protein level of SDF-1α, though this protein was excluded in initial array screening (Fig. 3b–d). Our findings suggest that both chemokines secreted from hPSCs themselves and feeder cells are the main supernatant components in hPSC culture.

### Migration of hPSCs functionally relies on chemokine signals

We further examined the mRNA levels of the CC and CXC subfamilies of chemokine receptors in the hPSCs. Quantitative PCR showed that chemokine receptors were expressed in the hPSCs and demonstrated variable mRNA levels (Fig. S10). It is worth noting that IL-8 receptors CXCR1/2 and SDF-1α receptor CXCR4 had higher mRNA expressions in the hESCs than in the feeder cells (Fig. S10a). Conversely, IP-10 receptor CXCR3 and SDF-1α receptor CXCR7 exhibited lower expressions in hESCs than in feeder cells. In addition, a considerable difference was found in mRNA levels between hESCs and hiPSCs, though both were cultured in mTeSR1 (Fig. S10b).

**Figure 4:**
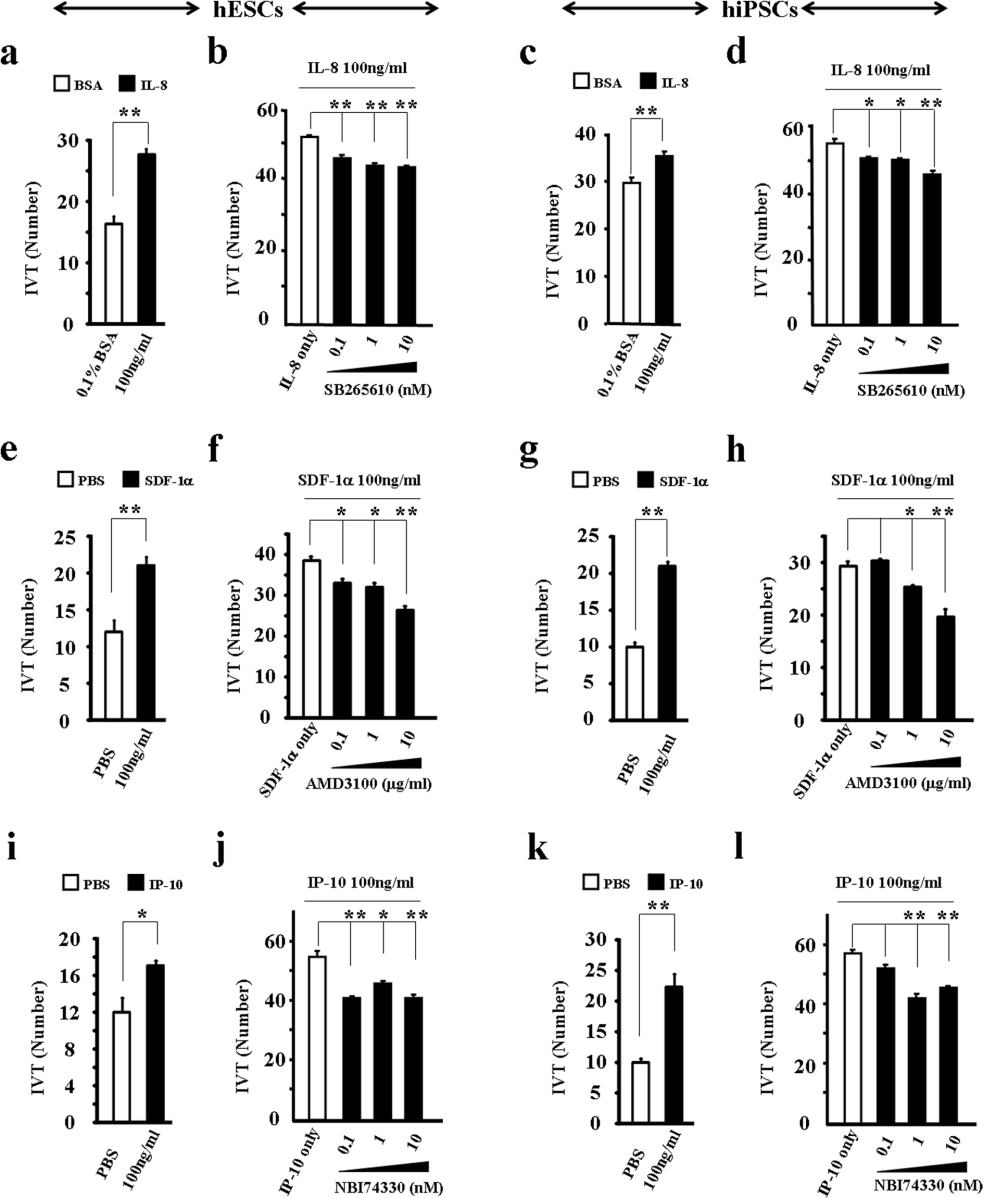
Chemokine signaling functionally mediates the transmigration of hPSCs. Chemoattractant effect of exogenous IP-10, IL-8, and SDF-1α on hPSCs. hESCs (**a**,**b**,**e**,**f**,**i**,**j**); hiPSCs (**c**,**d**,**g**,**h**,**k**,**l**); IL-8 (**a**,**c**) and CXCR2 antagonist SB265610 (**b**,**d**); SDF-1α (**e**,**g**) and CXCR4 antagonist AMD3100 (**f**,**h**); IP-10 (**i**,**k**) and CXCR3 antagonist NBI74330 (**j**,**l**). Values on graphs represent means ± sem, n = 3 individual experiments. *P < 0.05, **P < 0.01.

The broad expression of chemokine receptors in hPSCs indicates that chemokines might mediate their migration. To address this hypothesis, we tested the chemoattractant effects of IL-8, IP-10, and SDF-1α. We found that these three human recombinant proteins functioned as exogenous sources to induce the efficient transmigration of both hESCs and hiPSCs *in vitro* (Fig. 4). For instance, migrated hESCs and hiPSCs increased by 69.4% and 19.1%, respectively, after treatment with 100 ng/ml of IL-8 (Fig. 4a and c). However, the IL-8-induced transmigration of hESCs and hiPSCs significantly decreased after treatment with CXCR2-specific inhibitor SB265610 (Fig. 4b and d). Similar observations were obtained after examining the effects of SDF-1α, IP-10, and their receptor antagonists (Fig. 4e–l). Moreover, cell growth images and MTT assay demonstrated no toxicity or side effects of the antagonists on hESC survival (Fig. S11a–g). Similarly, these antagonists had no side effects on hiPSC survival (Fig. S11h–n). These results suggest that chemokine signals functionally mediate the migration of hPSCs.

### Maintenance of hPSCs depends on chemokine signaling

**Figure 5:**
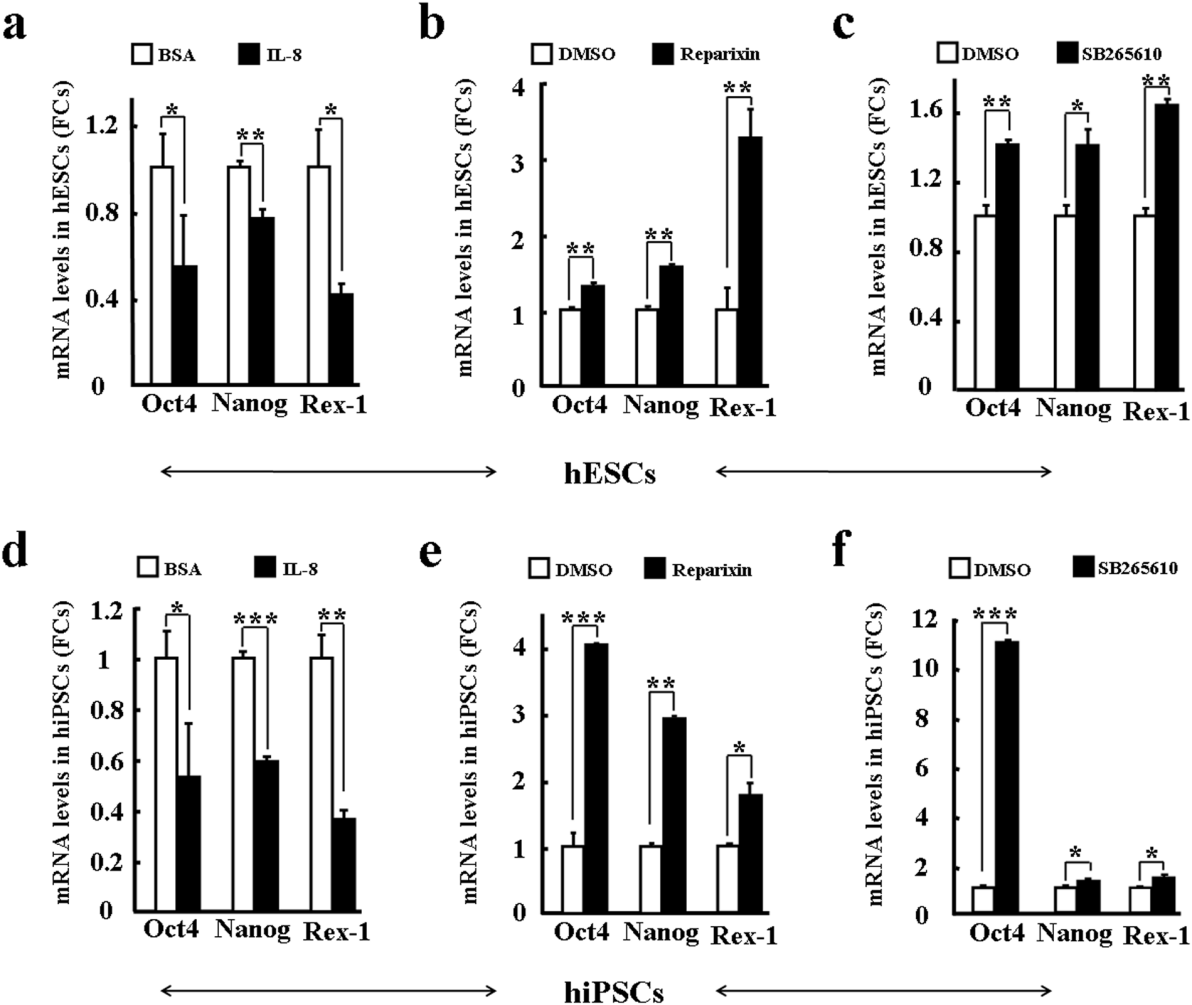
Chemokine IL-8 facilitates the differentiation of hPSCs. Stemness of hPSCs was evaluated by the expressions of three pluripotency genes (*Oct4*, *Nanog*, and *Rex-1*) after treatment with IL-8 and CXCR2 antagonists reparixin/SB265610. hESCs (**a**–**c**); hiPSCs (**d**–**f**); IL-8 (**a**,**d**); Reparixin (**b**,**e**); and SB265610 (**c**,**f**). Values on graphs represent means ± sem, n = 3 individual experiments. *P < 0.05, **P < 0.01, ***P < 0. 001.

Previous studies suggest that chemokines are important signals for maintaining tissue-specific stem cells. We hypothesized that hESC-secreting or feeder cell-secreting chemokines have similar roles on hPSCs as on tissue-specific stem cells. To test this, quantitative PCR was used to examine the mRNA expressions of *Oct4* and *Nanog* in hPSCs after treatment with chemokines or their receptor inhibitors. We found that exogenous IL-8 significantly decreased mRNA expressions of *Oct4*, *Nanog*, and *Rex-1* in hESCs (Fig. 5a). However, blocking IL-8 receptors CXCR1/2 with antagonists reparixin and SB265610 increased the mRNA expressions of *Oct4*, *Nanog*, and *Rex-1* in hESCs (Fig. 5b and c), with similar observations obtained for hiPSCs (Fig. 5d–f). Importantly, quantitative PCR of three differentiation genes (*AFP*, *c-actin*, and *Sox1*) demonstrated that exogenous IL-8 significantly increased the mRNA expressions of *AFP*, *c-actin*, and *Sox1* in hPSCs (Fig. S12a,d), whereas blocking IL-8-CXCR1/2 signaling with reparixin/SB265610 decreased the mRNA expressions (Fig. S12b,c,e,f). These results strongly suggest that IL-8 and its signaling pathway might differentiate hPSCs.

**Figure 6:**
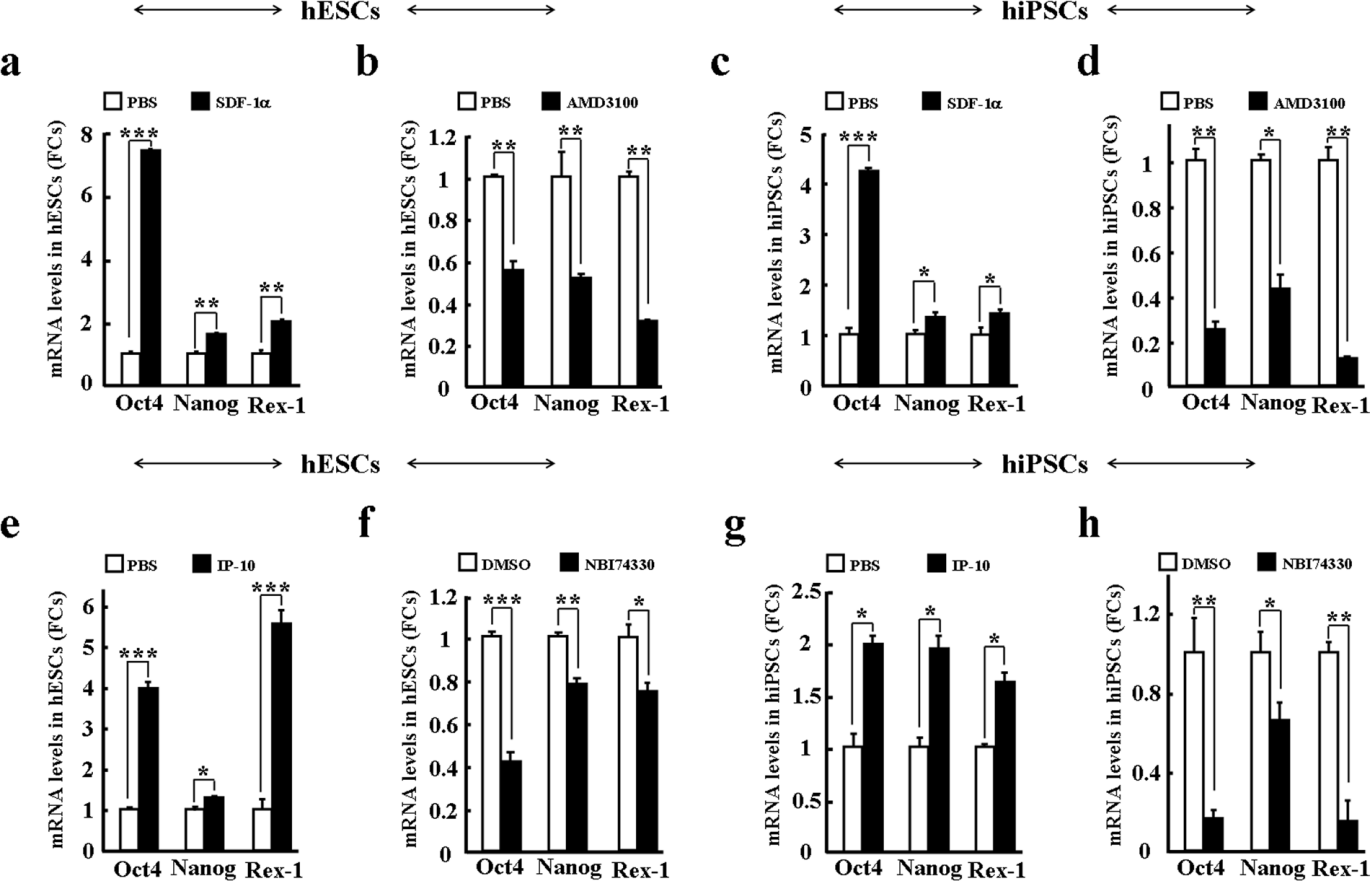
Chemokines SDF-1α and IP-10 enhance the stemness of hPSCs *in vitro*. Stemness of hPSCs was evaluated by the expressions of three pluripotency genes (*Oct4*, *Nanog*, and *Rex-1*) after treatment with agonists and antagonists. hESCs (**a**,**b**,**e**,**f**); hiPSCs (**c**,**d**,**g**,**h**); SDF-1α (**a**,**c**); CXCR4 antagonist AMD3100 (**b**,**d**); IP-10 (**e**,**g**); and CXCR3 antagonist NBI74330 (**f**,**h**). Values on graphs represent means ± sem, n = 3 individual experiments. *P < 0.05, **P < 0.01, ***P < 0.001.

We also examined the effects of exogenous SDF-1α on the maintenance of hPSCs. Different from IL-8, we found that SDF-1α tended to enhance the mRNA expressions of *Oct4*, *Nanog*, and *Rex-1* in hESCs and hiPSCs, respectively (Fig. 6a and c). These observations were confirmed by blocking the SDF-1α receptor CXCR4 with AMD3100, with mRNA levels of *Oct4*, *Nanog*, and *Rex-1* significantly decreased in both hESCs and hiPSCs (Fig. 6b and d). Furthermore, exogenous SDF-1α significantly decreased the mRNA expressions of *AFP*, *c-actin*, and *Sox1* in hPSCs (Fig. S13a,c), but blocking SDF-1α-CXCR4 signaling with AMD3100 increased their mRNA expressions (Fig. S13b,d). In addition, IP-10 was found to enhance the mRNA expressions of *Oct4*, *Nanog*, and *Rex-1*, but reduce the mRNA expressions of *AFP*, *c-actin*, and *Sox1* in hPSCs (Figs 6e–h and S13e–h). The above findings strongly suggest that supernatant chemokines are essential for maintaining the stemness of hPSCs, with chemokines IL-8, IP-10, and SDF-1α possibly playing a critical role in regulating the maintenance of hPSCs.

## Discussion

Human pluripotent stem cells include both hESCs and hiPSCs, which are characterized by self-renewal and pluripotency reflecting their potential differentiation capability^60^. Under *in vitro* culture, these unique cells can be maintained long-term in either KSR or mTeSR1 media without loss of their stemness. In the present study, to prevent potential differentiation, inactivated HFFs were provided as feeder cells in KSR culture or were replaced by coating culture plates with 1% Matrigel in mTeSR1 culture^61^. So far, intracellular molecules involved in the long-term maintenance of hPSC stemness have been widely characterized^62^. Unfortunately, chemokines and their underlying signaling pathways have not yet been systematically studied, although these small secretion molecules represent the major cytokine portion in cell cultures. The large chemokine superfamily is generally responsible for cellular migration, proliferation, and survival^18,21^. Recently, increasing evidence suggests that chemokines are essential intracellular signals that help maintain the stemness of multipotent tissue-specific stem cells^8,63^. However, the extent of chemokines and their impact on hPSC stemness during long-term *in vitro* culture remain poorly understood. To explore the possible roles of chemokines related to hPSCs, we established a new hESC cell line by isolating and culturing ICM cells from fertilized human eggs. These hESCs not only expressed stem cell markers Oct4, Nanog, TRA-1-60, and TRA-1-81, but also showed potential pluripotency. For instance, hESC-derived EBs highly expressed the three germ layer markers through differentiation *in vitro*, with these hESCs able to form the normal germ layer tissues through transplantation into immunodeficient mice. Like hiPSCs, these hESCs could be maintained long-term in both KSR and mTeSR1 media. Furthermore, both hESCs and hiPSCs expressed pluripotency markers Oct4, TRA-1-81, Nanog, and TRA-1-60.

**Figure 7:**
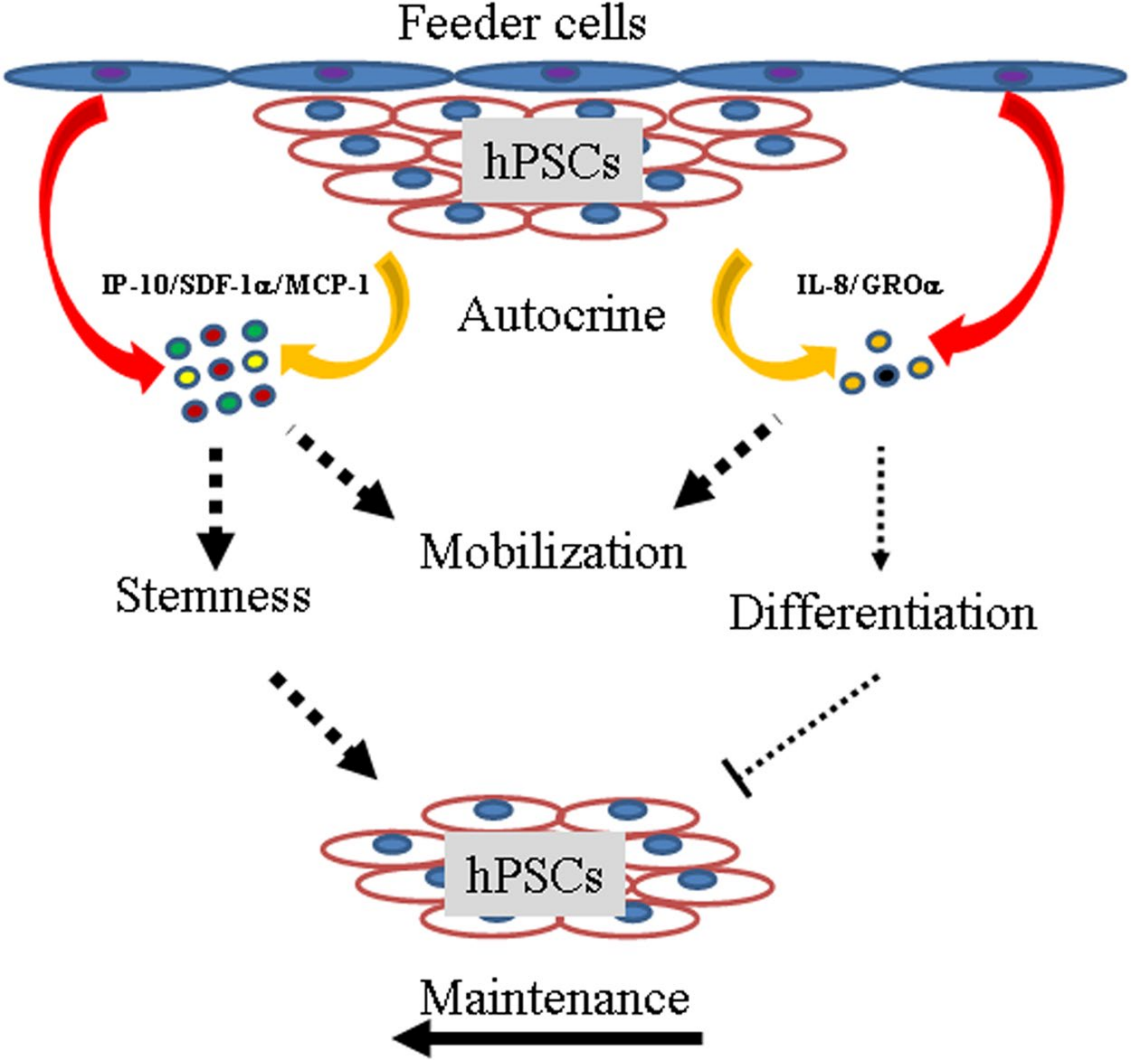
Mobility and maintenance of hPSCs require multiple chemokine signals. Schematic representation of predominant chemokines from feeder cells or hPSCs themselves. 1) Released chemokines uniformly mediate the migration of hPSCs; 2) Both differentiating and maintaining chemokines were secreted in the culture supernatant. However, the final effect of chemokines tended to maintain the stemness of hPSCs *in vitro*.

To determine how many chemokines were in the hPSC culture, we further applied protein arrays to screen the supernatant chemokine components of the hESCs and hiPSCs. In total, 38 chemokines were included in the array, consisting of 61% of CC, 35% of CXC, 2% of XC, and 2% of CX3C subfamilies, respectively (Table 1). Except for CXC chemokine SDF-1α, 37 chemokines showed detectable probing signals. However, the failed detection of SDF-1α might be due to its relatively low concentration since high protein concentrations of SDF-1α were detected by ELISA. Through high-throughput screening, 30 and 18 chemokines were identified from the hESCs and hiPSCs under the mTeSR1 conditions, respectively. Interestingly, 25 chemokines were directly detected from the mTeSR1 medium. However, chemokines secreted from both hiPSCs and mTeSR1 were lower than their counterparts secreted from hESCs. Under KSR culture conditions, indirect evidence indicated that 29 chemokines were secreted from hESCs. Similarly, both the feeder cells and KSR medium might provide extra chemokines. These results suggest that secretions from hPSCs and feeder cells equally contributed to the final chemokine cocktail (Fig. 7), with chemokines GROα, GRO, IL-8, GCP-2, and IP-10 demonstrating the highest supernatant concentrations in the hESC culture. In summary, our findings support the hypothesis that chemokines are important cytokines for hPSCs based on their secretion spectra and protein concentrations.

Chemokines provide conserved chemoattractant functions for nearly all cell types. More importantly, chemokines mediate the migration of multipotent stem cells^30,43,49,56,63^. In the current study, we examined the chemotactic effect of three representative chemokines, that is, IL-8, SDF-1α, and IP-10. These three chemokines demonstrated very uniform induction of both hESC and hiPSC migration. Blocking their corresponding cognate receptors on hPSCs significantly reduced ligand-induced *in vitro* transmigration. This offers further evidence to support that chemokines and their receptors provide signaling to mediate the mobilization of hPSCs.

Microenvironment (niche)-dependent SDF-1α has been shown to enhance the maintenance of hematopoietic stem cells (HSCs) as well as other tissue-specific stem cells^25^. Recent studies also suggest that GROα and MCP-1 mediate the pluripotency of hPSCs^53–55^. For instance, GROα-CXCR2 chemotaxis has been reported to enhance the differentiation of hESCs to adopt a neuronal fate^54^. Conversely, it has also been suggested that IL-8 and GROα signaling through CXCR2 might support both pluripotency and proliferation of hPSCs without exogenous bFGF^58^. We observed that IL-8-CXCR1/2 decreased the pluripotency of hPSCs; for example, IL-8-CXCR1/2 decreased the expressions of pluripotency markers (*Oct4*, *Nanog*, and *Rex-1*), but increased the expressions of differentiation markers (*AFP*, *c-actin*, and *Sox1*). However, this signaling regulation was not necessary for the proliferation of hPSCs (Fig. S11). This evidence suggests that some chemokines, represented by IL-8 and GROα, participate in the differentiation of hPSCs. In addition, MCP-1 and CCR2 reportedly support pluripotency of both human and mouse iPSCs^55,57^. In the current study, we identified that SDF-1α-CXCR4 and IP-10-CXCR3 play similar roles in enhancing the pluripotency of hPSCs. Again, we did not observe an obvious effect of SDF-1α on the proliferation of hPSCs (Fig. S11). However, NBI 74330 (a CXCR3 antagonist) did inhibit the proliferation of hPSCs (Fig. S11g,n), though the potential regulatory mechanism involved in this process needs to be further studied. These findings suggest that other chemokines represented by MCP-1, SDF-1α, or IP-10 enhance the stemness of hPSCs. Therefore, we hypothesize that chemokines play two different roles to regulate the maintenance of hPSCs. The final maintenance of hPSCs depends not only on balanced chemokine secretion, but also on signaling shifts that tend to increase their pluripotency (Fig. 7).

## Materials and Methods

### Ethics statement

All experiments were carried out according to the relevant guidelines. All human sample studies and protocols were reviewed and approved by the Ethics Committee of the First People’s Hospital of Yunnan Province (AMEP: 2016009) and the Life Sciences Department of Yunnan University, China. Experiments were conducted following the exact regulations issued by both committees. For all human samples, legal informed consent was provided by all donors and patients.

### Human specimens

Human foreskin tissue was provided by the Department of Urology from Kunming Children’s Hospital. Human blastocysts were obtained from the Department of Reproduction and Genetics of the First People’s Hospital of Yunnan Province. The final developmental stage of human blastocysts was strictly limited to post-fertilization day 6. Legal informed consent was provided by all donors and patients.

### Establishment of HFFs and hESCs

Human foreskin tissue was digested by 0.25% trypsin and cultured in Dulbecco’s modified Eagle’s medium (DMEM) with 10% defined fetal bovine serum (FBS) to obtain human foreskin fibroblasts (HFFs). To prepare feeder cells, HFFs were further inactivated by 10 μg/ml of mitomycin C (Sigma, USA). Human blastocysts were washed in PBS and then treated with 0.25% pronase (Sigma, USA) to remove zona pellucid. The inner cell masses (ICMs) were next dissected from the trophectoderm by immunosurgery with normal anti-human and anti-guinea pig whole serum (Jackson Immune Research, USA). Isolated ICMs were directly plated into Nunc 4-well plates with HFFs as the feeder cells and further cultured in modified KSR medium. The DMEM/F12 medium included 20% knockout serum replacement (KSR), 0.1 mM MEM non-essential amino acid, 0.1 mM 2-mercaptoethanol, and 8 ng/ml of basic fibroblast growth factor. All other reagents were purchased from Life Science Inc. (Gibco, USA). ESC-derived clones were continuously passaged for at least 10 generations to establish stable embryonic stem cell lines.

### *In vitro* culture and treatment of hPSCs

The p27 hiPSC cell line hNF C11 was purchased from the Guangzhou Institute of Biomedicine and Health, Chinese Academy of Sciences. Both newly-established hESCs and hiPSCs were seeded in 6-well plates pre-coated with 1% Matrigel (BD Biosciences) and cultured with the mTeSR1 complete kit (Stem Cell Technology, USA). Recombinant human chemokine proteins IL-8, SDF-1α, and IP-10 were purchased from R&D Systems (USA). CXCR1/CXCR2-specific antagonist reparixin and CXCR3-specific antagonist NBI74330 were bought from MCE (USA). CXCR4-specific antagonist AMD3100 and CXCR2-specific antagonist SB265610 were purchased from Sigma (USA). The hPSCs were treated by either 1 μg/ml of IL-8, 100 nM reparixin, 100 nM SB265610, 100 ng/ml of SDF-1α, 100 nM AMD3100, 50 ng/ml of IP-10, or 100 nM NBI74330 overnight.

### Quantitative PCR

The mRNA expressions of chemokine cognate receptors, three germ layer markers, and pluripotency markers *Oct4* and *Nanog* in hPSCs were examined by quantitative PCR (primers provided in Table S1) performed on an ABI 7300 sequence detection instrument (Applied Biosystems, USA) following the manufacturer’s instructions. Total RNA was extracted from human ESCs and iPSCs using total RNA isolation reagent (TaKaRa, Japan). The first-strand cDNA was synthesized using Oligo (dT) 18 primer (PrimeScript® 286 RT, TaKaRa) per the manufacturer’s protocols. Quantitative PCR was conducted following standard procedures using SYBR® 303 Premix Ex Taq (TaKaRa, Japan). Sequences of oligo primers are listed in Table S1. All quantitative PCR experiments were independently repeated three times.

### Immunofluorescence staining

Both hESCs and hiPSCs were fixed in 4% phosphate-buffered paraformaldehyde (PFA). Immunofluorescence staining was carried out using standard procedures. Briefly, fixed hESCs and hiPSCs were stained by rabbit anti-human Oct4, rabbit anti-human Nanog, mouse anti-human TRA-1-81, and mouse anti-TRA-1-60 antibodies. Goat secondary antibodies anti-rabbit conjugated with Alex594, anti-mouse conjugated with Alex 488, and anti-rat conjugated with Alex 594 were chosen. TRA-1-81/TRA-1-60 primary antibodies and secondary antibodies were purchased from Millipore (USA). Primary antibodies AFP, GTATA4, desmin, actin, nestin, and β-III tubulin were purchased from Wanleibio (China).

### Alkaline phosphatase staining

Alkaline phosphatase staining was conducted using an alkaline phosphatase assay kit (Beyotime Biotechnology, China) according to the standard procedures described by the manufacturer.

### Embryoid body differentiation

Human ESC clumps (~500 cells) were collected and suspended in KSR medium without bFGF to form embryoid bodies (EBs). The culture medium was replaced with DFSR medium after 5 d of culture. The EBs were further differentiated in DFSR medium (KSR medium without bFGF, but supplemented with 10% FBS) on Matrigel for 10–15 d. In the EBs, mRNA expressions of germ layer markers *AFP*, *GATA4*, *GATA6*, *Sox17*, and *FOXA2* (endoderm), *T*, *MIXL1*, *TBX1*, and *c-actin* (mesoderm), and *Sox1*, *Nestin*, *NeuroD1*, and *PAX6* (ectoderm) were examined by quantitative PCR. In addition, germ layer protein markers AFP and GATA4 (endoderm), desmin and actin (mesoderm), and nestin and β-III tubulin (ectoderm) were evaluated by immunofluorescence staining.

### Karyotyping

G-band karyotyping was conducted in the Genetics and Diagnosis Laboratory from the First People’s Hospital of Yunnan Province. Briefly, hESCs were treated with 0.6 mg/ml of colchicine (Gibco, USA) for 1.5 h. Next, hypotonic hESCs were prepared after treatment with 0.075 mol/L potassium chloride for 20 min in a 37 °C water bath. Hypotonic hESCs were fixed in methanol and glacial acetic acid mixture (3:1). Chromosomal spreads were obtained by dropping prepared hESCs directly onto glass slides. Giemsa staining of human chromosomes was conducted according to standard protocols.

### Teratoma formation assay

The hESCs were cultured in a 6-well plate with mTeSR1 medium on 1% Matrigel. Six healthy female NOD SCID mice (6–8 weeks old) were purchased from Charles River Laboratories (USA). Human ESC clumps (~3 × 10^6^ cells) were mixed with 0.5 g/ml Matrigel and injected intramuscularly into the NOD SCID mice. After 6–8 weeks, teratomas were observed, surgically excised, and fixed in 4% PFA overnight. Teratoma tissues were embedded in paraffin, sliced into 5-µm sections, and stained by hematoxylin and eosin according to standard protocols.

### Imaging

Fluorescent, phase contrast, and bright-field pictures were taken using a Leica DMi8 automated and manual microscope (Leica, Germany). Pictures were analyzed by Leica Application Suite X software (Leica, Germany). Karyotypes were scanned by the CytoVision scanning system of an Olympus Exol microscope (Olympus, Japan).

### Chemokine array

Supernatants were collected and screened using Ray-Bio Human Chemokine Antibody Array C1 kits (RayBiotech, USA) according to the standard protocols provided by the manufacturer. FluorChem E (Protein Simple, USA) was used to detect signal chemiluminescence intensities from protein array membranes. Quantitative analysis for chemiluminescent intensity was conducted using NIH image J Version 1.43. Relative signal intensity (RSI) was calculated by subtracting chemiluminescence intensities with negative control (NC) intensities. A RSI-based heatmap was constructed using R statistical software. Hierarchical clustering algorithm with correlation dissimilarity measure was applied to group sample RSIs in red-green color-coded grids to represent high and low RSIs, respectively.

### Enzyme-linked immunosorbent assay (ELISA)

Supernatants were collected after 48 h of cell culture. We purchased ELISA kits for detecting human IL-8 (CXCL8) (Cusabio Biotech, China), IP-10 (CXCL10) (Shanghai Enzyme-Linked Biotech Co., Ltd, China), and SDF-1α (CXCL12) (Elisa Biotech Co., Ltd, China). ELISA experiments were conducted following the manufacturers’ instructions. Plates were read by a micro-plate reader (SpectraMax®314 340PC, USA) at a wave length of 450 nm.

### *In vitro* transmigration

*In vitro* transmigration was performed using 48-well AP48 Boyden chambers (Neuro Probe, USA) according to the methods provided by the manufacturer. Briefly, 30 μl of 0.1% BSA (control) and different concentrations of recombinant human chemokine proteins were added to the lower chambers. The lower chambers were separated with 8-μm pore-sized polycarbonate membranes (Neuro Probe, USA). Then, 50 μl of cell suspension (~40,000 cells per ml) was seeded in the top wells. To block chemokine-induced transmigration, receptor-specific antagonists were added to the top wells. Boyden chambers were incubated at 37 °C with 5% CO_2_ for 4 h. After staining with 20 nM Hoechst 33342 (Thermo, USA), migrated cells on the filters were counted using an inverted fluorescence microscope (Olympus IX71, Japan). In addition, 0.1–10 nM SB265610, 0.1–10 µg/ml of AMD3100, and 0.1–10 nM NBI74330 were used to treat the cells. All transmigration experiments were independently repeated three times.

### MTT assay

A MTT (3-(4, 5-dimethyl-2-thiazolyl)-2, 5-diphenyl-2-H-tetrazolium bromide) assay was conducted according to the standard procedures described by the manufacturer’s instructions. Briefly, human ESC or iPSC clumps (~500 cells) were collected and seeded into 96-well plates (~1 × 10^4^ cells/well) in mTeSR1 medium on 1% Matrigel. The toxicities of 0.1–100 nM antagonists, including reparixin, SB265610, AMD3100, and NBI 74330, were tested according to the standard procedures described by the manufacturer. The plate was read using a micro-plate reader (SpectraMax®314 340PC, USA) at a wave length of 490 nm. The MTT solution (Cell Titer 96® Aqueous One Solution Reagent) was purchased from Program (USA).

### Statistics

All experiments were conducted in duplicate and data were expressed as means ± sem (standard error of mean). Statistical analyses were performed by one-way analysis of variance (ANOVA) with Dunnett’s test. Values of P < 0.05 were considered statistically significant.

## Electronic supplementary material

Supplementary information
