# Supplementary material for: Protein profiling identified key chemokines that regulate the maintenance of human pluripotent stem cells
Source: Sci Rep. 2017 Nov 6;7:14510. doi: 10.1038/s41598-017-15081-6 (PMC5674019; doi:10.1038/s41598-017-15081-6)
Supplement: Supplementary file 1 — Supplementary information [file 41598_2017_15081_MOESM1_ESM.pdf]

Protein profiling identified key chemokines that regulate the maintenance of human pluripotent stem cells

Zongmin Jiang[1,2*], Yonggang Li[3*], Xinglai Ji[2,4], Yiyuli Tang[2,4], Haijing Yu[1,2], Lei Ding[1,2], Min Yu[1,2], Qinghua Cui[1,2], Ming Zhang[5], Yanping Ma[3#], Meizhang Li[1,2#]

[1]Laboratory of Biochemistry and Molecular Biology, School of Life Sciences, Yunnan University, Kunming, Yunnan 650091, CHINA.

[2]Key Laboratory of Molecular Cancer Biology, Yunnan Education Department, Kunming, Yunnan 650091, CHINA

[3]Department of Reproduction and Genetics, the First People's Hospital of Yunnan Province, Kunming, Yunnan 650032, CHINA.

[4]State Key Laboratory for Conservation and Utilization of Bio-Resources in Yunnan, Yunnan University, Kunming, Yunnan 650091, CHINA.

[5]Yunnan Key Laboratory of Stem Cell and Regenerative Medicine, Institute of Molecular and Clinical Medicine, Kunming Medical University, Kunming, Yunnan 650500, CHINA

*Equal contribution

#Correspondence to: Yanping MA, Email: mayanpinghome@163.com. Department of Reproduction and Genetics, First People's Hospital of Yunnan Province, Kunming, Yunnan 650032, China; Or Meizhang LI, Email: mzhli@ynu.edu.cn. Laboratory of Biochemical and Molecular Biology, School of Life Sciences, Yunnan University, Kunming, Yunnan 650091, China.

Supplemental figures

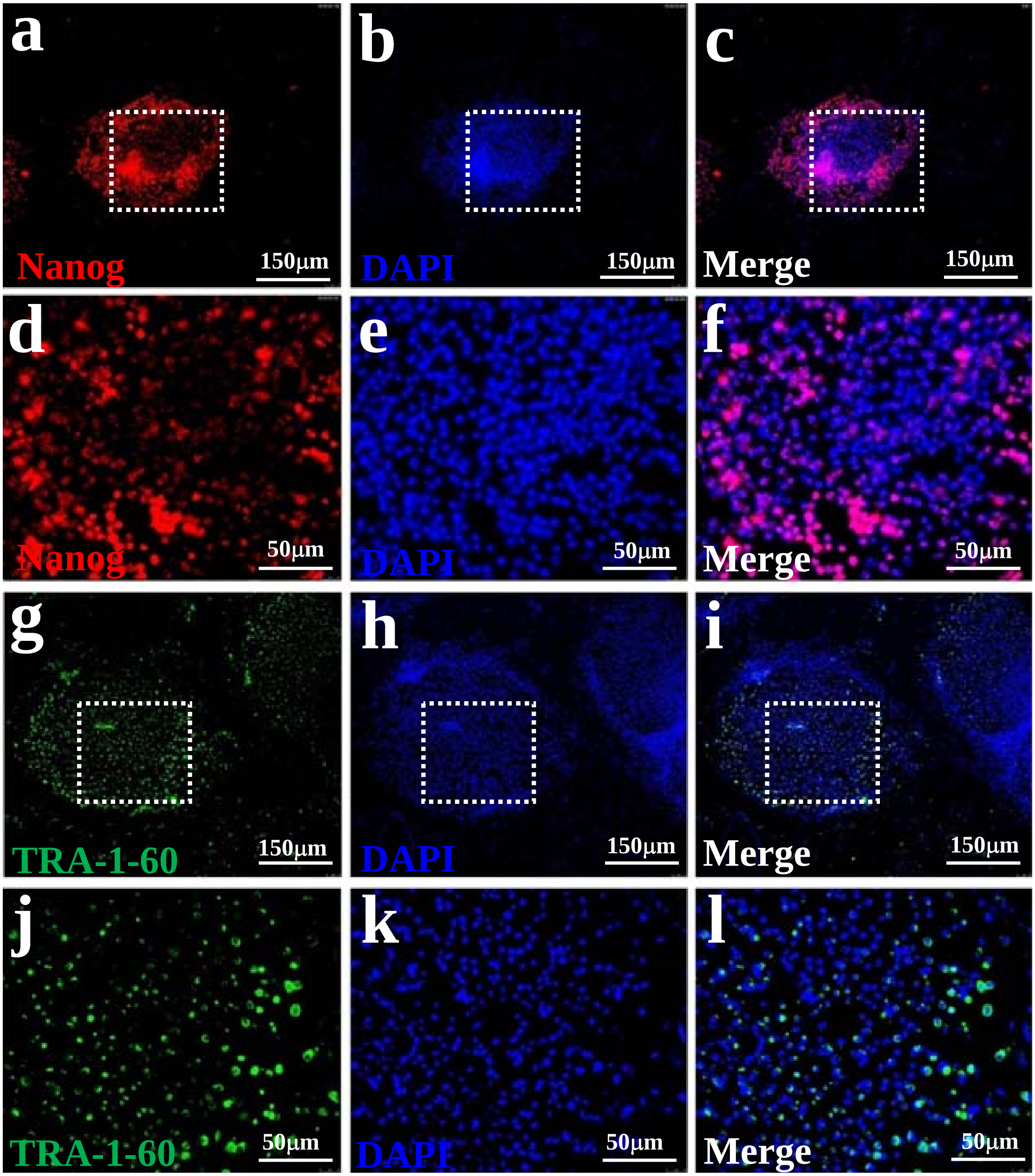

Figure S1. Protein expressions of Nanog and TRA-1-60 on hESCs cultured on feeder layers in KSR medium. (a-l) Immunostaining of pluripotency markers Nanog and TRA-1-60 in hESCs-derived clones. Nanog (**a**, **d**); TRA-1-60 (**g**, **j**); DAPI (**b**, **e**, **h**, **k**); and Merge (**c**, **f**, **i**, **l**). Scale bars, 150 µm (**a-c**, **g-i**) and 50 µm (**d-f**, **j-l**).

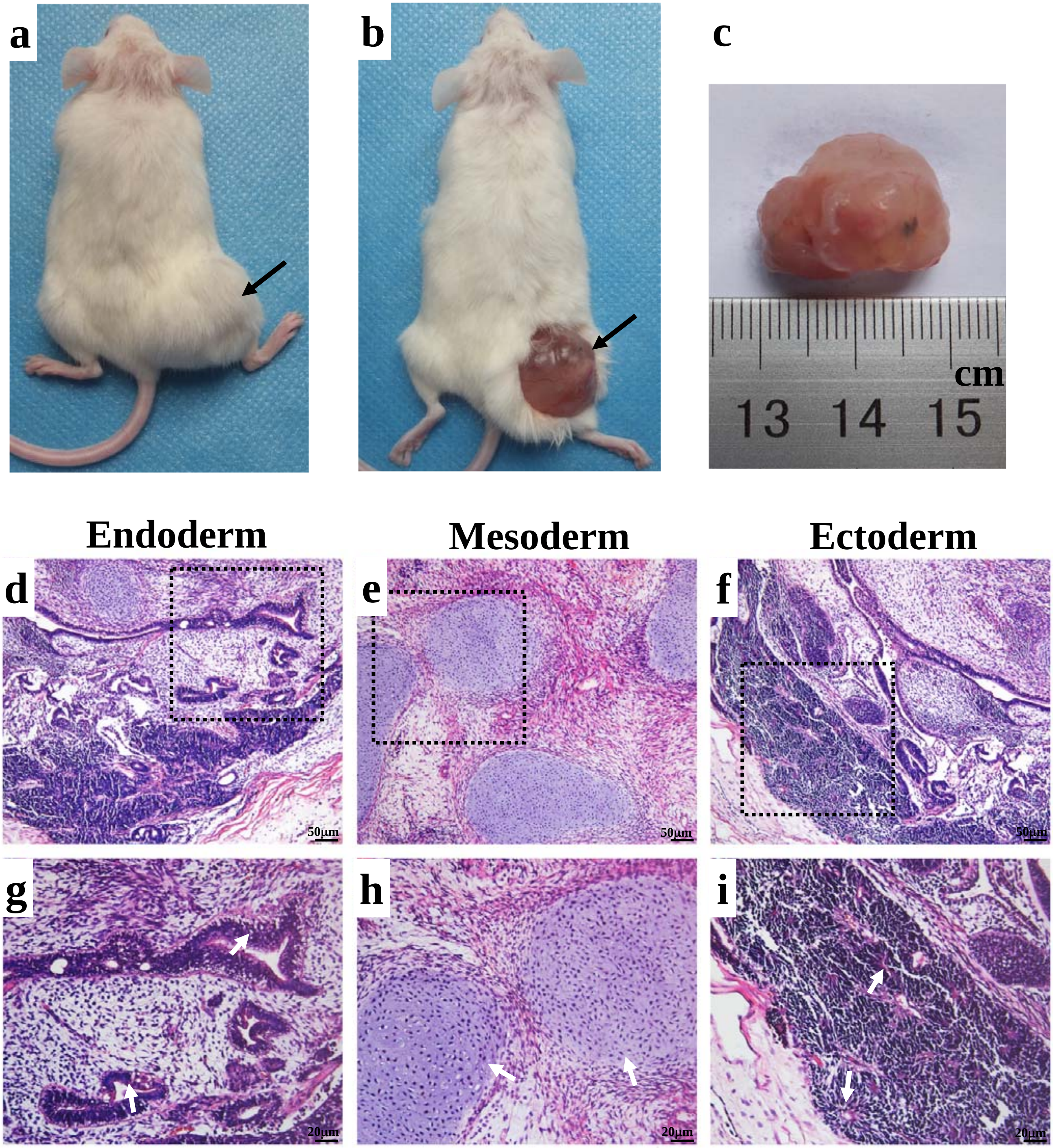

Figure S2. Differentiation of three germ layers of hESCs *in vivo*. (**a-c**) Teratomas (arrows) formed after hESCs were subcutaneously transplanted into NOD SCID mice for seven weeks. (**d-h**) Histopathological analysis of three germ layers of teratoma derived from hESCs by hematoxylin and eosin staining. Endoderm (**d**, **g**); mesoderm (**e**, **h**); ectoderm (**f**, **i**). Scale bars, 50 μm (**d**, **e**, **f**) and 20 μm (**g**, **h**, **i**).

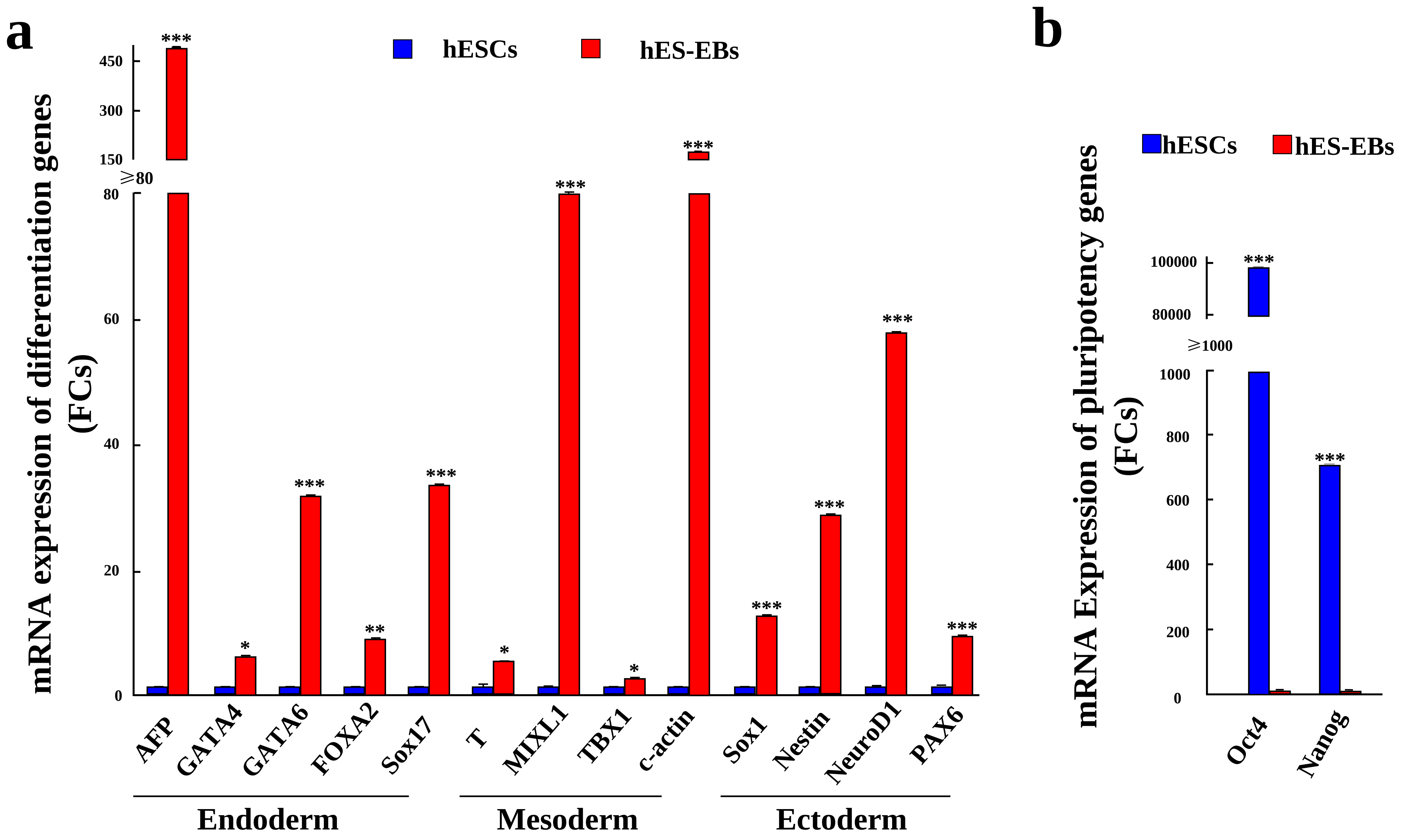

Figure S3. Quantitative PCR analysis of three germ layers and pluripotency gene markers in hESC-derived embryonic bodies (EBs). (**a**) *AFP*, *GATA4*, *GATA6*, *FOXA2*, and *Sox17* (endoderm); *T*, *MIXL1*, *TBX1*, and *c-actin* (mesoderm); *Sox1*, *Nestin*, *NeuroD1*, and *PAX6* (ectoderm). (**b**) *Oct4* and *Nanog*. Values on graphs represent means ± sem, n ≧ 3 individual experiments. *P < 0.05, **P < 0.01, ***P < 0.001.

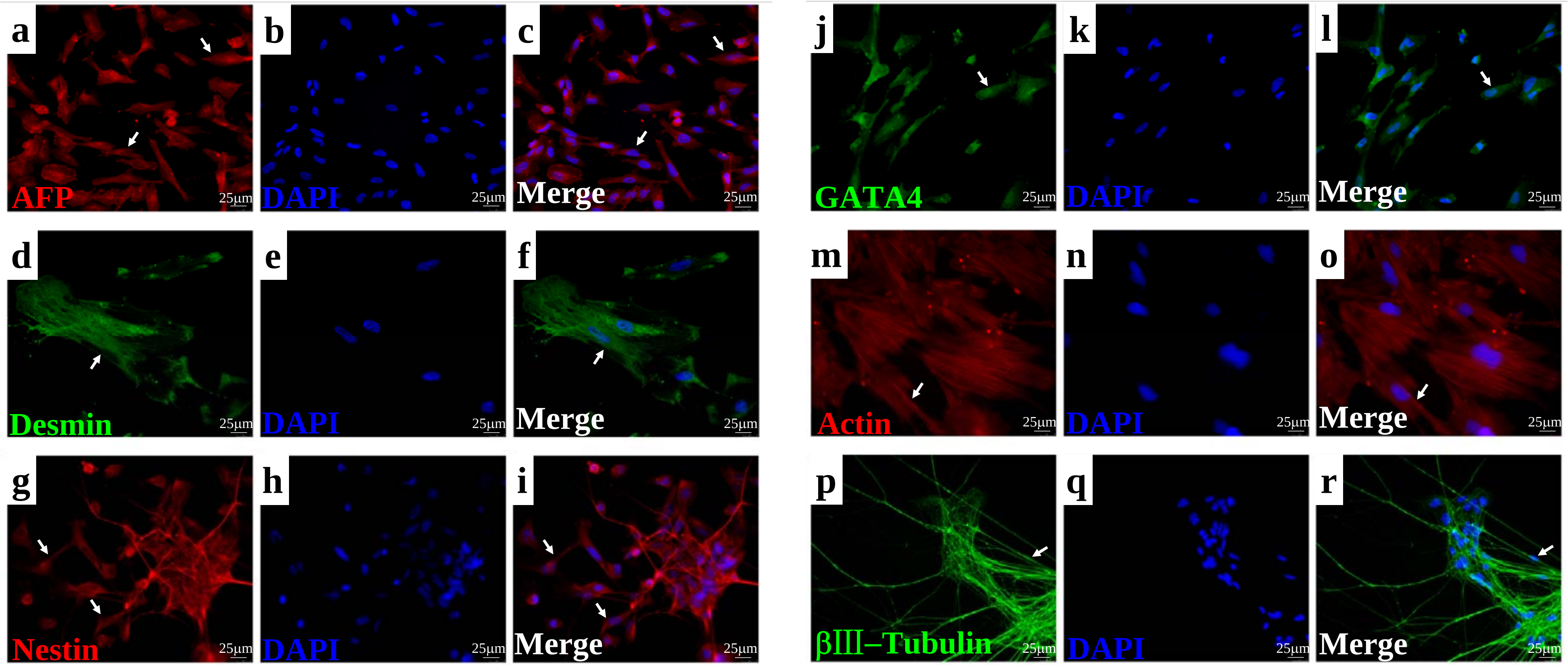

Figure S4. Immunostaining of three germ layer protein markers in EBs. (**a-r**) Endoderm (AFP, **a-c** and GATA4, **j-l**); Ectoderm (desmin, **d-f** and actin, **m-o**); Ectoderm (nestin, **g-i** and β-III tubulin, **p-r**). Cells were counterstained by DAPI. Scale bar, 25 µm.

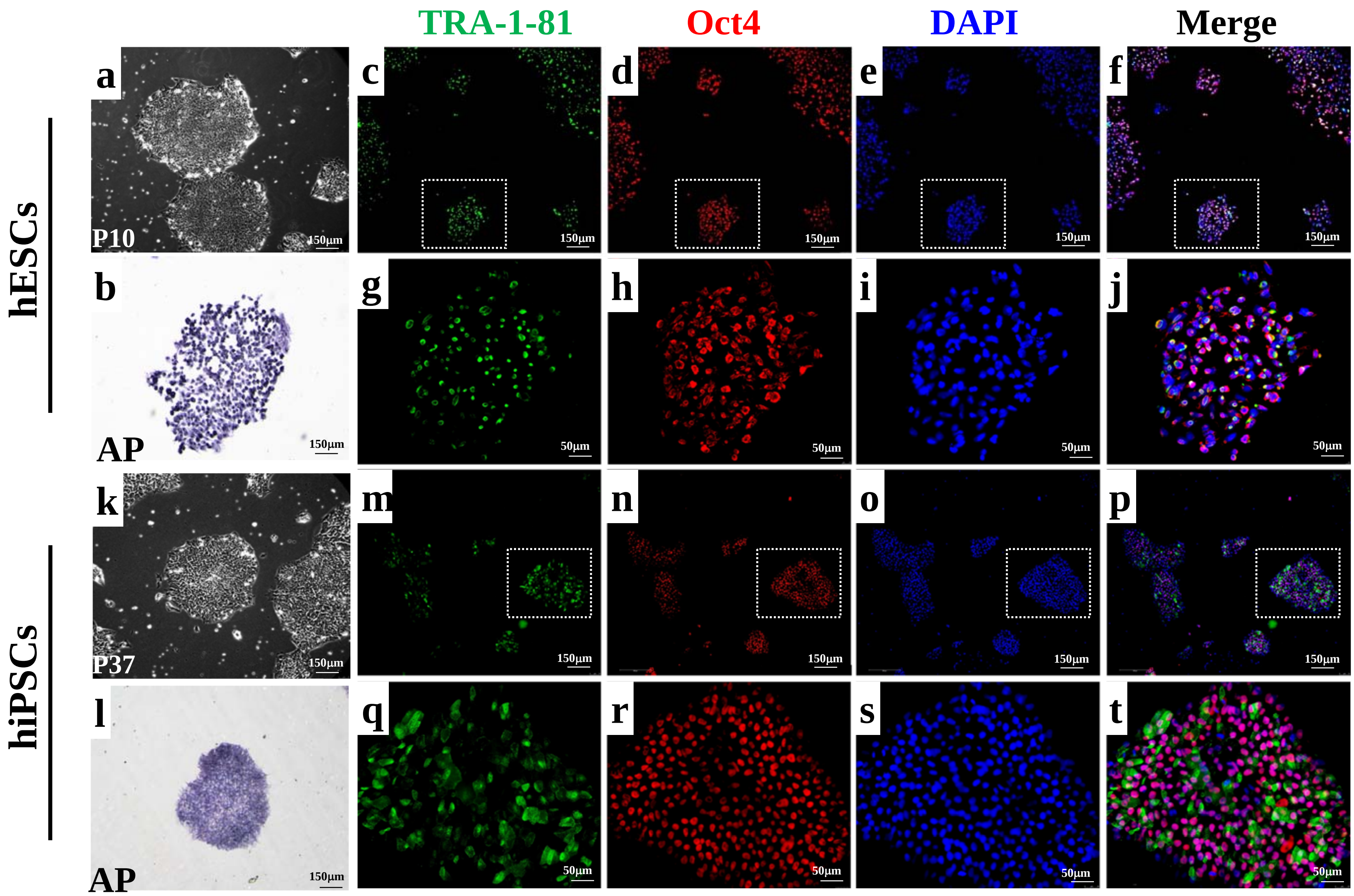

Figure S5. Maintenance of hESCs and hiPSCs in mTeSR1 medium. (a-j) hESC-derived clones. Scale bar, 150 mm. **(k-t)** hiPSC-derived clones. Both hESCs and hiPSCs were cultured in mTeSR1 medium. TRA-1-81 (**c**, **g**, **m**, **q**); Oct4 (**d**, **h**, **n**, **r**); DAPI (**e**, **i**, **o**, **s**); and Merge (**f**, **j**, **p**, **t**). Scale bars, 150 μm (**c-f**, **m-p**) and 50 μm (**g-j**, **q-t**).

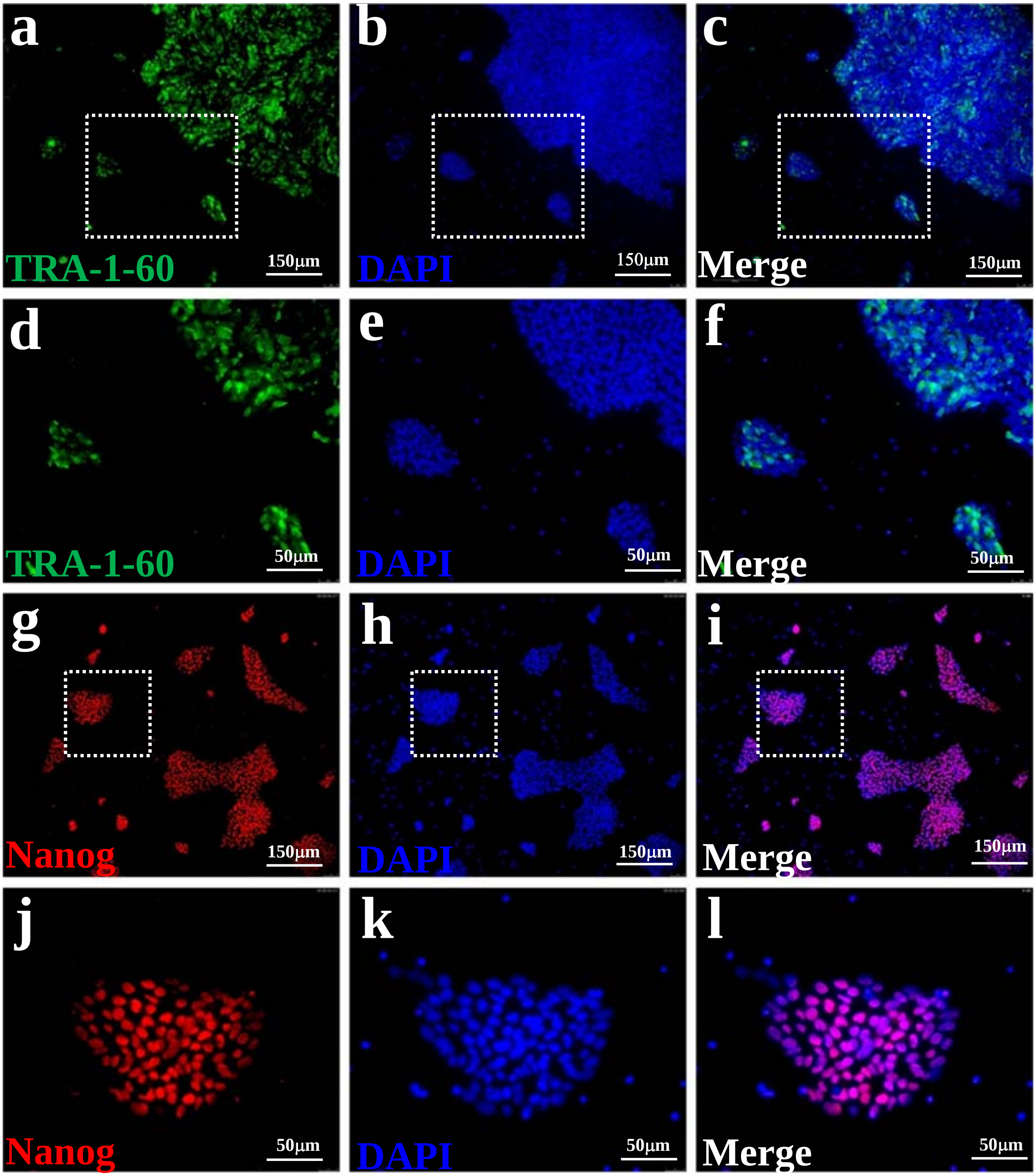

Figure S6. Protein expressions of Nanog and TRA-1-60 in hESCs cultured in mTeSR1 medium. (a-l) Immunostaining of pluripotency markers Nanog and TRA-1-60 in hESCs-derived clones. Nanog (**a**, **d**); TRA-1-60 (**g**, **j**); DAPI (**b**, **e**, **h**, **k**); and Merge (**c**, **f**, **i**, **l**). Scale bars, 150 µm (**a-c**, **g-i**) and 50 µm (**d-f**, **j-l**).

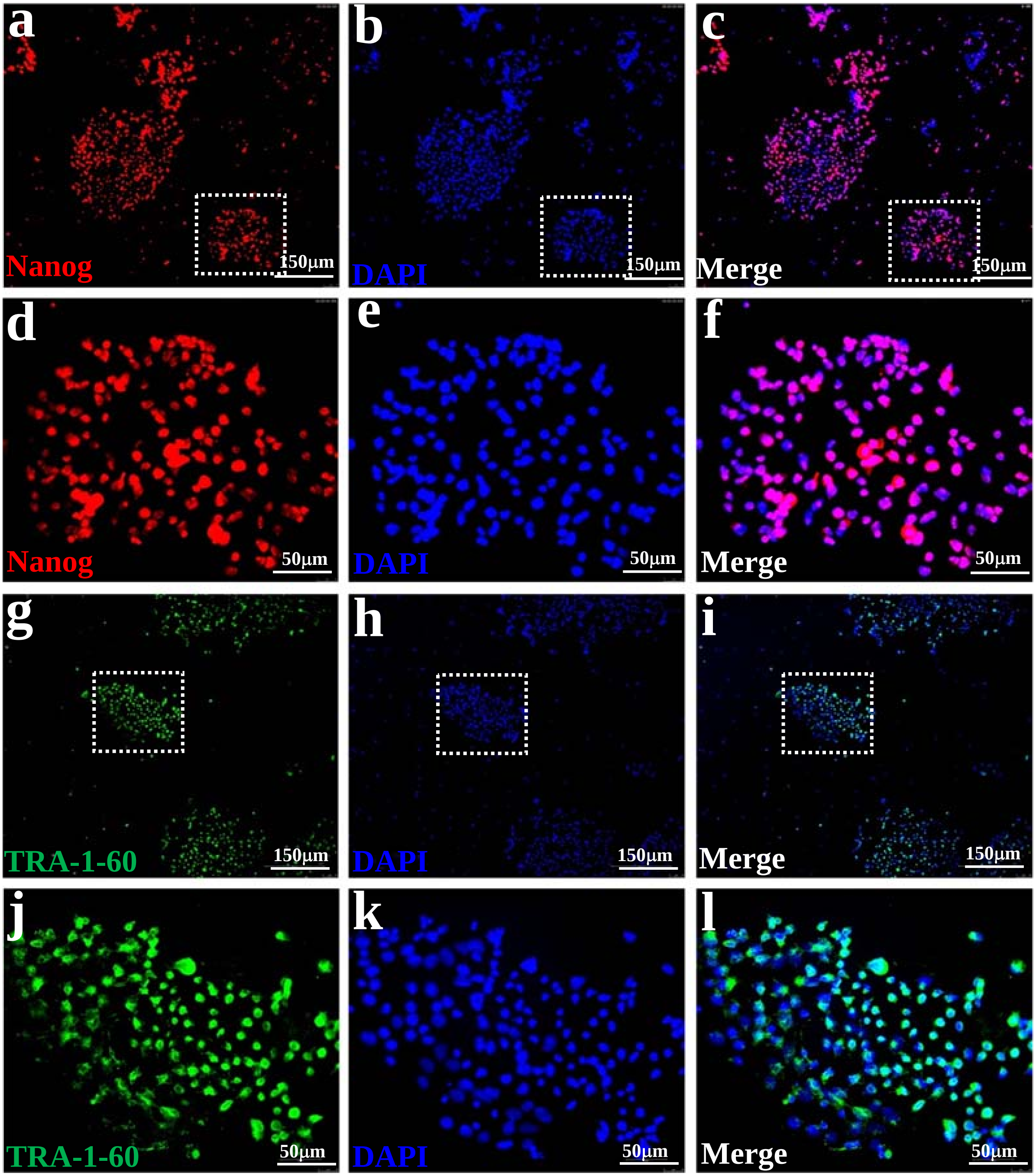

Figure S7. Protein expressions of Nanog and TRA-1-60 in hiPSCs cultured in mTeSR1 medium. (a-l) Immunostaining of pluripotency markers Nanog and TRA-1-60 in hESCs-derived clones. Nanog (**a**, **d**); TRA-1-60 (**g**, **j**); DAPI (**b**, **e**, **h**, **k**); and Merge (**c**, **f**, **i**, **l**). Scale bars, 150 μm (**a-c**, **g-i**) and 50 μm (**d-f**, **j-l**).

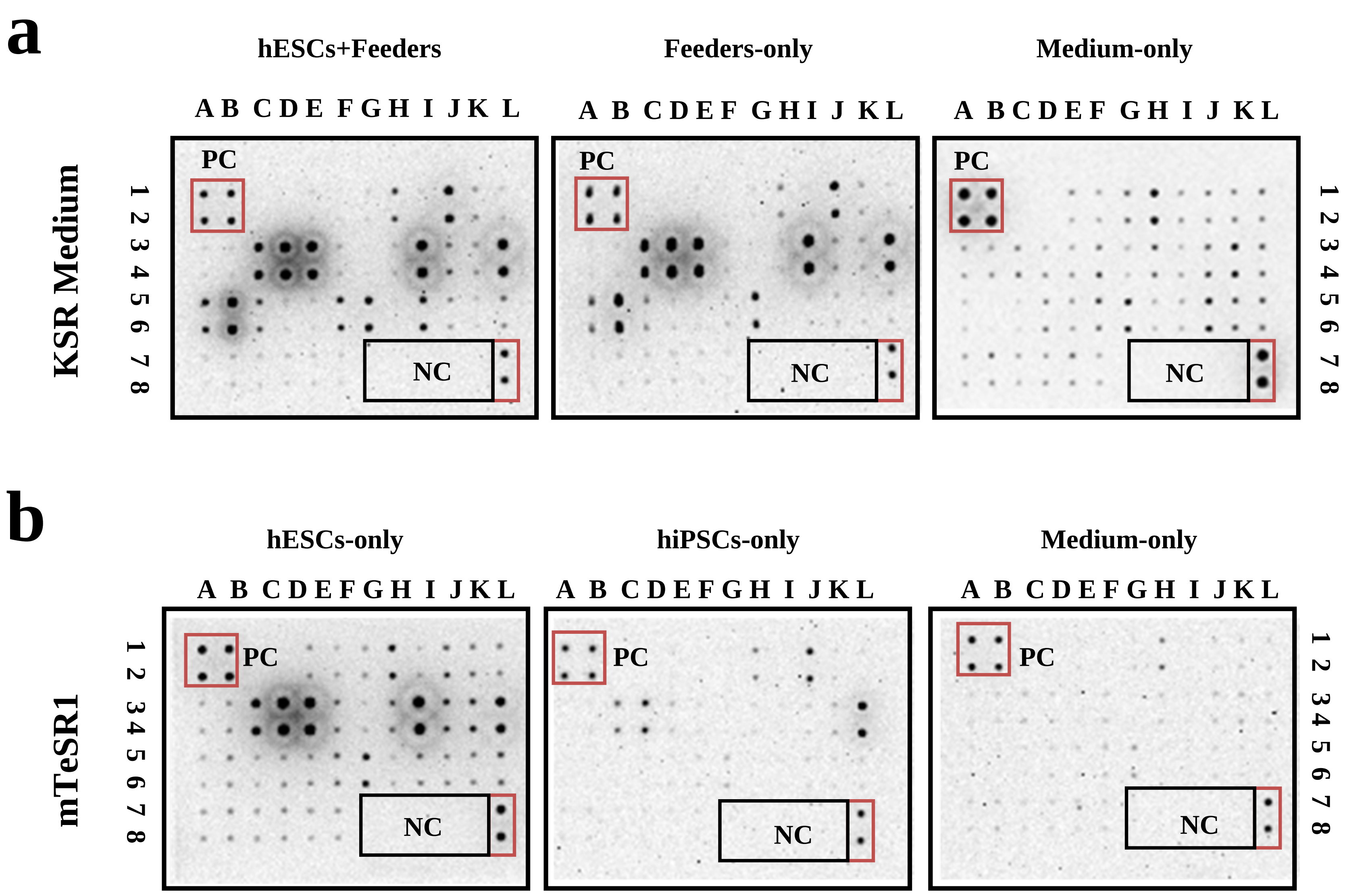

Figure S8. Signal intensities of chemokines in protein arrays. (a-b) Chemokines from six different supernatants were evaluated. KSR medium (**a**); mTeSR1 medium (**b**); Positive controls (PCs); Negative controls (NCs). Relative signal intensity (RSI) was defined as per the normalized grey intensities listed in Table 1.

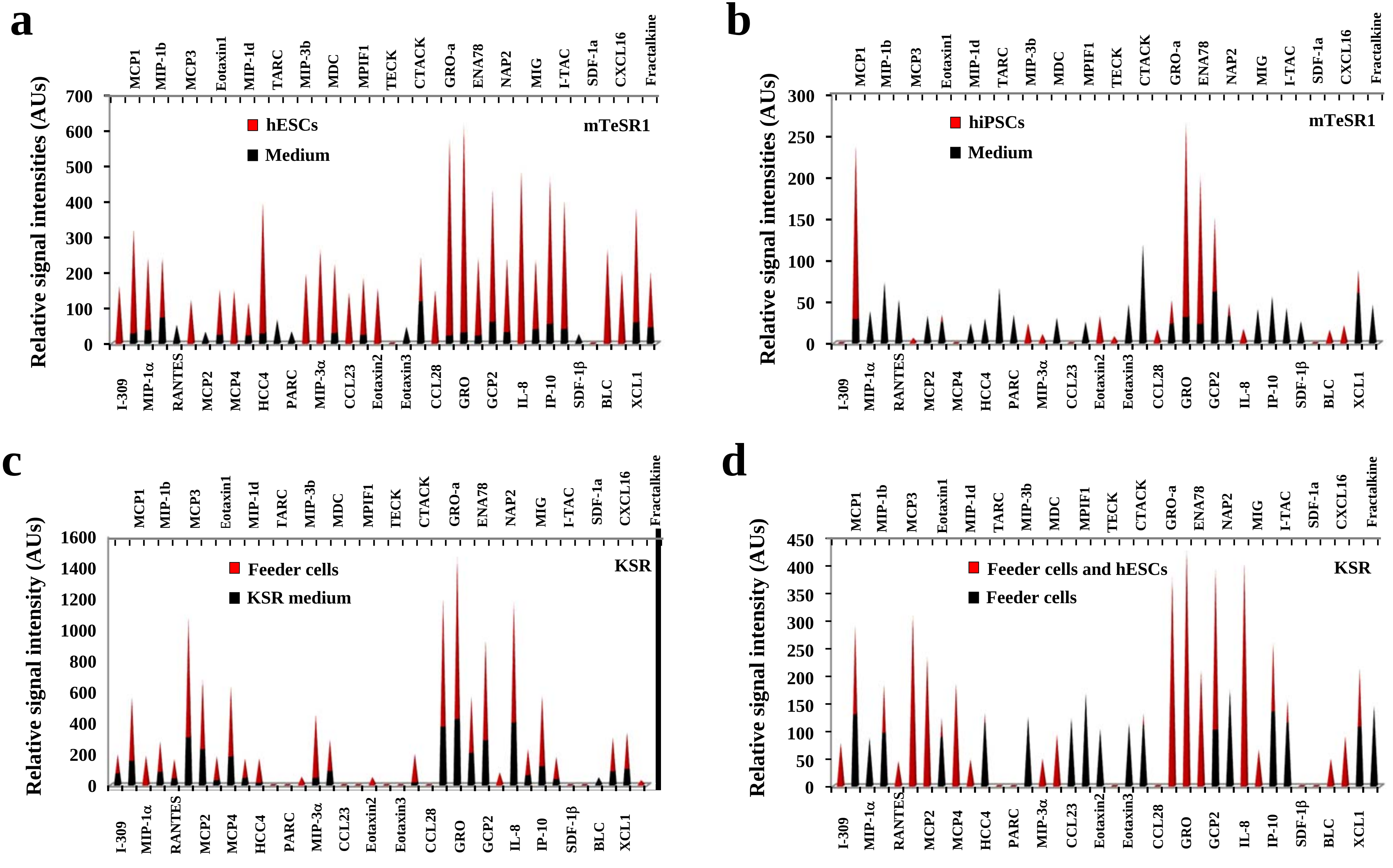

Figure S9. Comparison of chemokine secretion of hPSCs under different conditions. hESCs vs mTeSR1 medium (**a**); hiPSCs vs mTeSR1 medium (**b**); Feeder cells vs KSR medium (**c**); and Feeder cells/hESCs vs KSR medium (**d**). AUs, arbitrary units.

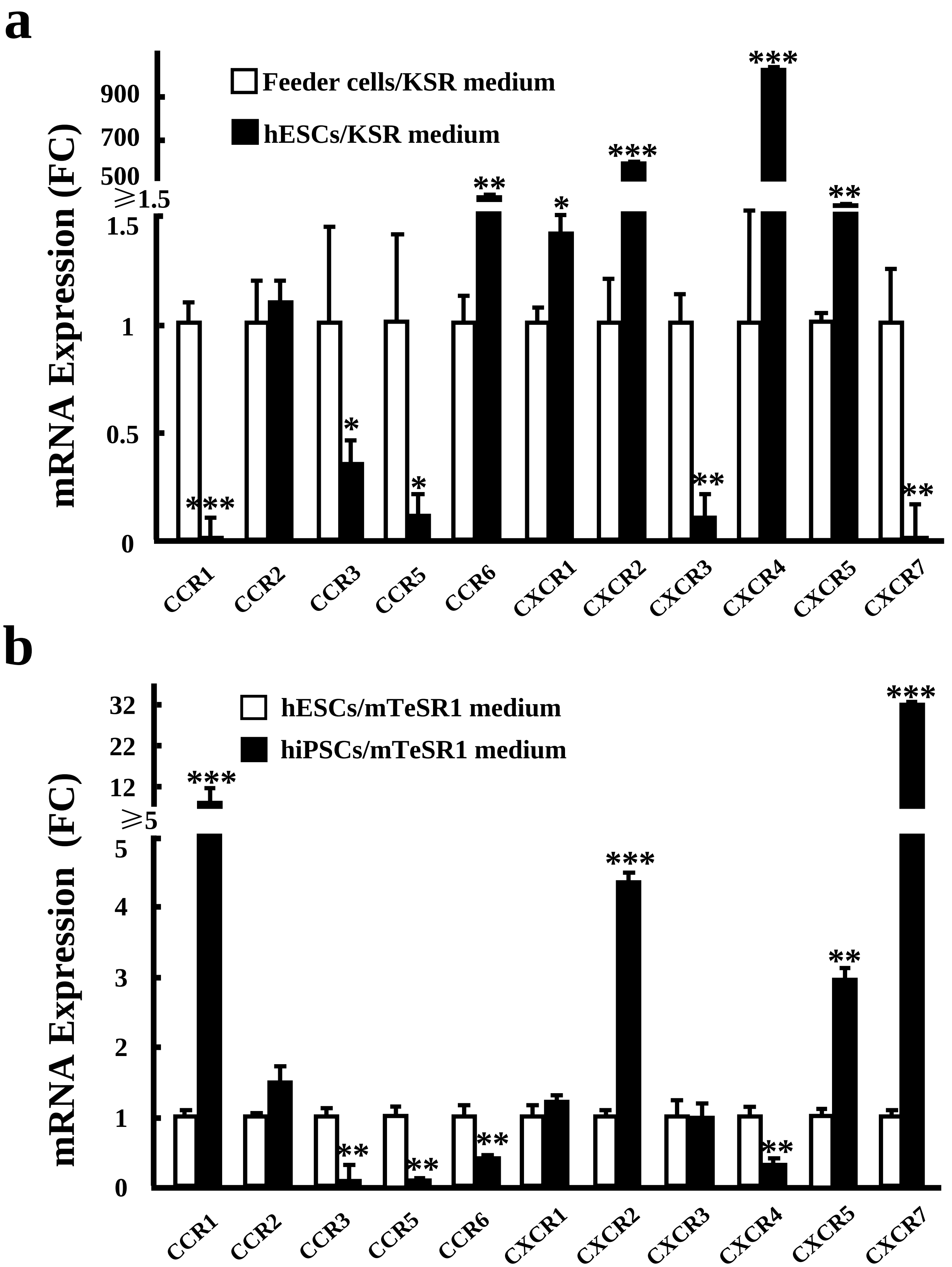

Figure S10. mRNA expression of CC/CXC chemokine receptors in hPSCs.

(a) Feeder cells vs hESCs in KSR medium; **(b)** hESCs vs hiPSCs in mTeSR1 medium. Values on graphs represent means ± sem; FC, fold change; n = 3 individual experiments. *P < 0.05, **P < 0.01, ***P < 0.001.

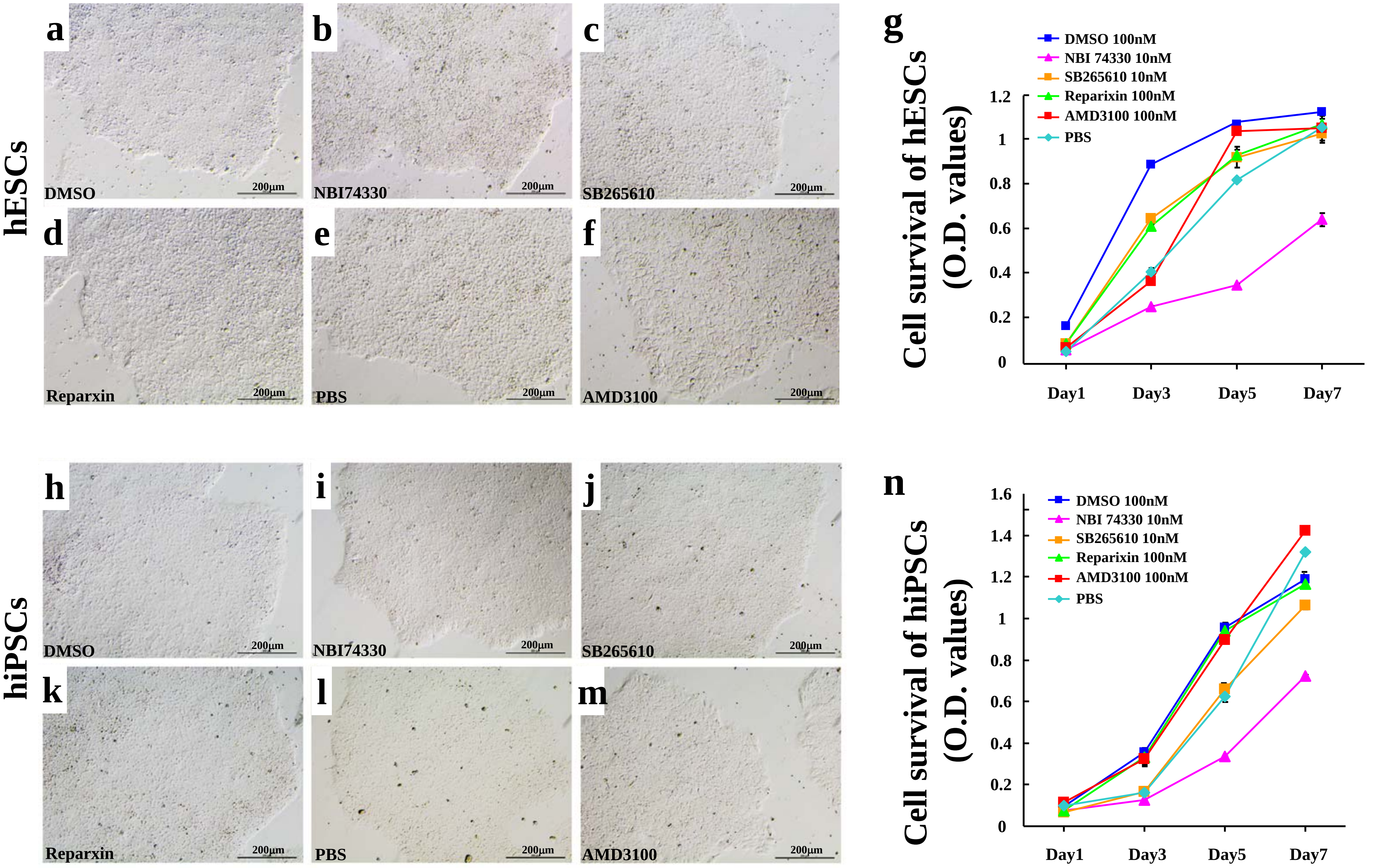

Figure S11. Effects of antagonists on both hESCs and hiPSCs. (**a-f**, **h-m**) Phase microscope images of hPSCs treated with several receptor-specific antagonists for 7 d. hESCs (**a-f**); hiPSCs (**h-m**). Scale bar, 200 µm. (**g**, **n**) MTT assay of hPSC survival and toxicity responses to receptor-specific antagonists. hESCs (**g**); hiPSCs (**n**).

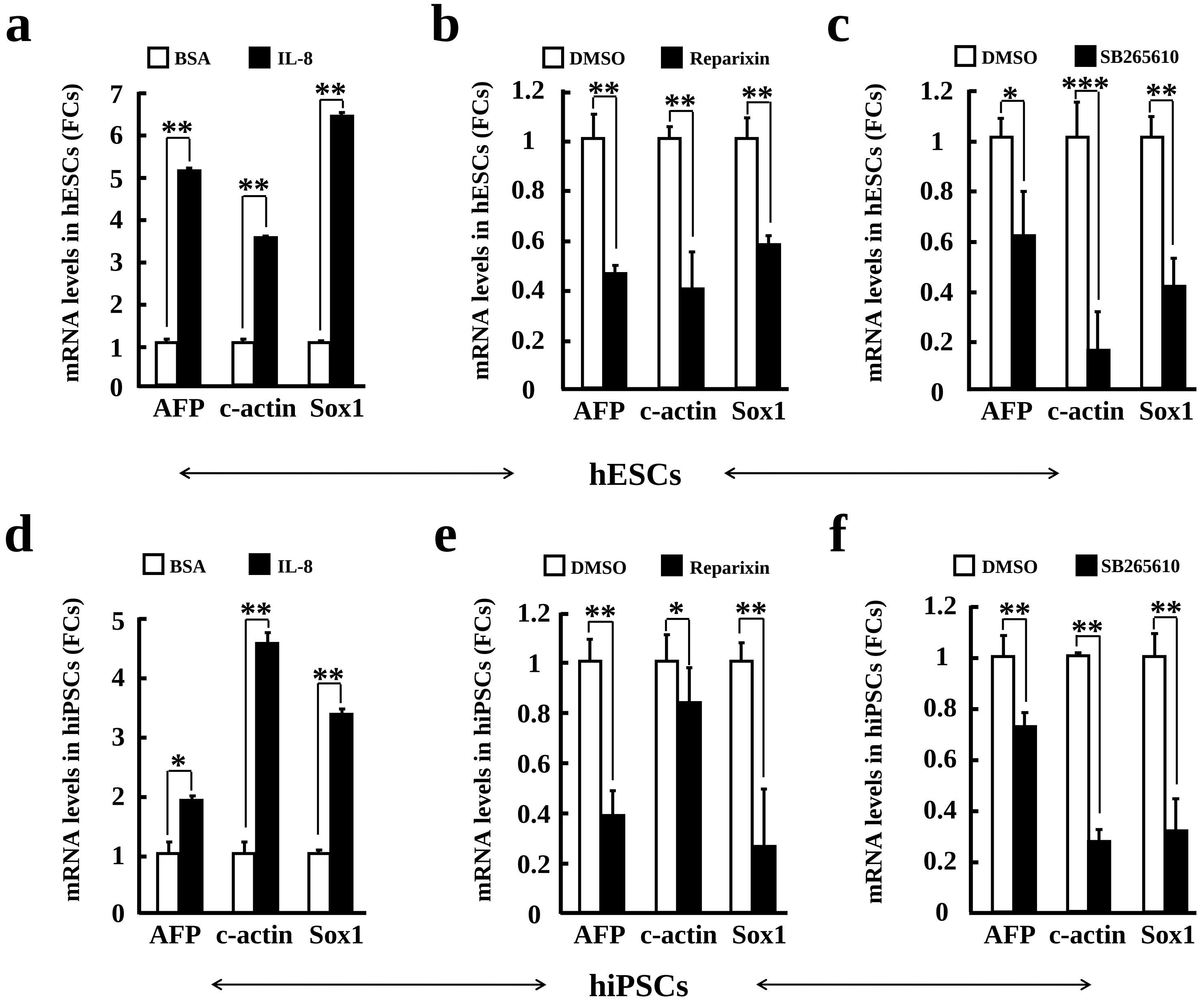

Figure S12. Chemokine IL-8 signaling in the *in vitro* differentiation of hESCs and hPSCs. Differentiation of hESCs (**a-c**) and hiPSCs (**d-f**) was evaluated by the expressions of three germ genes (*AFP*, *c-actin*, and *Sox1*) after treatment with IL-8 and CXCR2 antagonists reparixin/SB265610. hESCs (**a-c**); hiPSCs (**d-f**); IL-8 (**a**, **d**); Reparixin (**b**, **e**); and SB265610 (**c**, **f**). Values on graphs represent means ± sem, n ≧ 3 individual experiments. *P < 0.05, **P < 0.01, ***P < 0. 001.

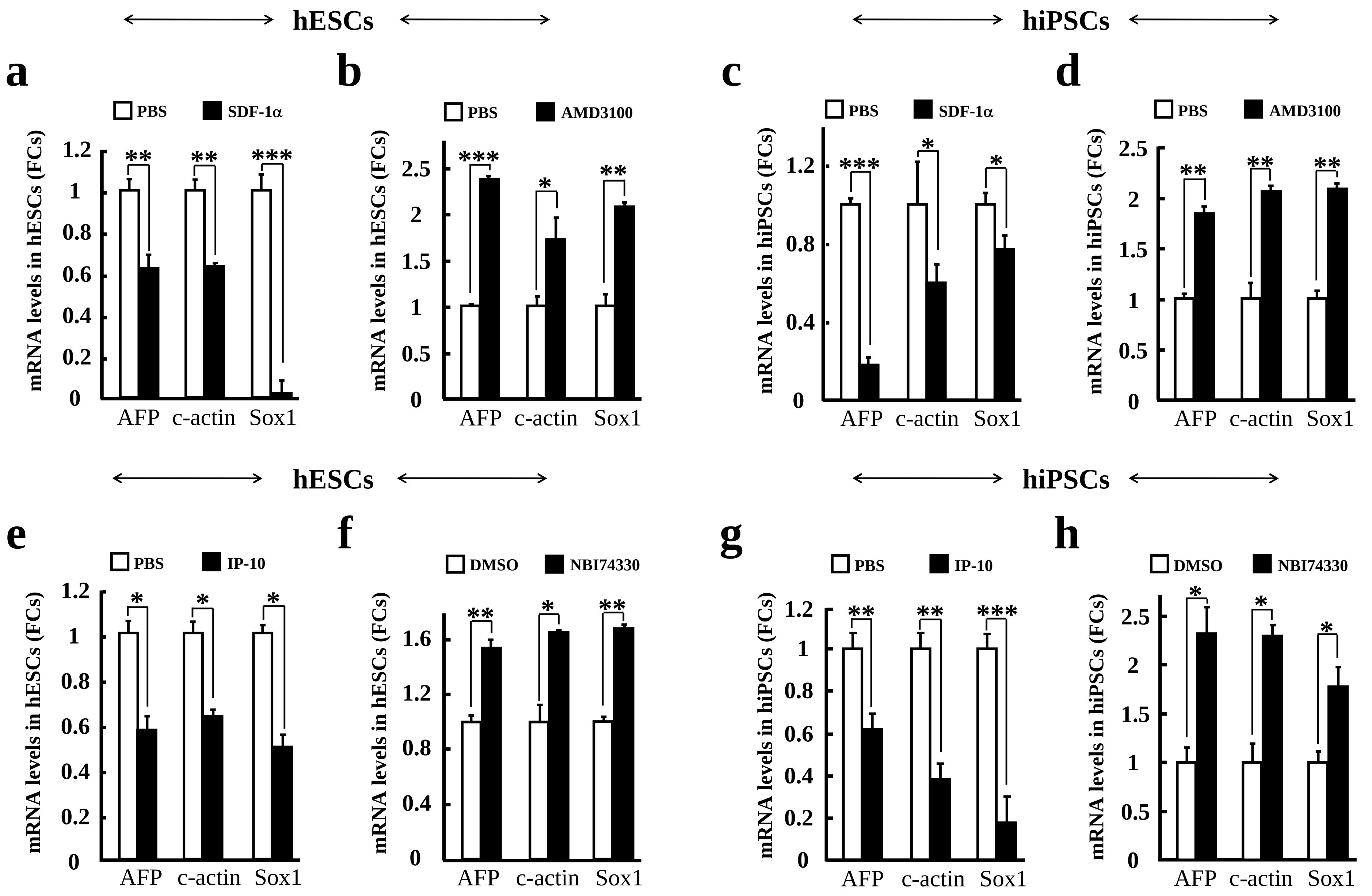

Figure S13. Chemokines SDF-1a and IP-10 in the *in vitro* differentiation of hESCs and hPSCs. Differentiation of hPSCs was evaluated by the expressions of three germ genes (*AFP*, *c-actin*, and *Sox1*) after treatment with AMD3100/NBI 74330. hESCs (**a**, **b**, **e**, **f**); hiPSCs (**c**, **d**, **g**, **h**); SDF-1a (**a**, **c**); CXCR4 antagonist AMD3100 (**b**, **d**); IP-10 (**e**, **g**); and CXCR3 antagonist NBI74330 (**f**, **h**). Values on graphs represent means ± sem, $n \geqq 3$ individual experiments. $^*P < 0.05$, $^{**}P < 0.01$, $^{***}P < 0.001$.

Supplementary table 1.

Oligo primers of chemokine receptors and transcription factors.

Name	Sequence
**Chemokine receptors**	
CCR1 forward	5'-GGCTGGTATTGCCTTTGTTG-3'
CCR1 reverse	5'-GGTCCAAATGTCTGCTCTGC-3'
CCR2 forward	5'-ACGGTGCTCCCTGTCATAAA-3'
CCR2 reverse	5'-CATTCCCAAAGACCCACTCAT-3'
CCR3 forward	5'-CTGTCTCGTTCTCCCTCTGCT-3'
CCR3 reverse	5'-GCTCCGCTCACAGTCATTTC-3'
CCR5 forward	5'-GGGGTGGTGACAAGTGTGAT-3'
CCR5 reverse	5'-GATGATTCCTGGGAGAGACG-3'
CCR6 forward	5'-AGGCAGTTCTCCAGGCTATTT-3'
CCR6 reverse	5'-GGCATTGCTGAAAACCCAC-3'
CXCR1 forward	5'-CCAGTCCAGTTTGCTATGAGG-3'
CXCR1 reverse	5'-AGCAGGACCAGGTTGTAGGG-3'
CXCR2 forward	5'-ACCTACTCTTTGCCCTGACCT-3'
CXCR2 reverse	5'-ACAGACCCCAGATGCTGAGAC-3'
CXCR3 forward	5'-ATCAACTTCTACGCAGGAGCC-3'
CXCR3 reverse	5'-AGGAAGATGAAGTCTGGGAGG-3'
CXCR4 forward	5'-TCTTTGTCATCACGCTTCCC-3'
CXCR4 reverse	5'-CGCCAACATAGACCACCTTT-3'
CXCR5 forward	5'-TGGGAACTGGACAGATTGGA-3'
CXCR5 reverse	5'-GTCTCCGTGGAACTGCGTGT-3'
CXCR7 forward	5'-CTTCCTTGTCTGCTGGTTGC-3'
CXCR7 reverse	5'-GCTTGGTGAGCCCTGTTTT-3'
**Transcription Factors**	
Oct4 forward	5'- AGCGACTATGCACAACGA-3'
Oct4 reverse	5'- AGTGGTGACGGAGACAGG-3'
Nanog forward	5'-ACCTATGCCTGTGATTTGTGG-3'
Nanog reverse	5'-CGGGACCTTGTCTTCCTTTT-3'
Rex-1 forward	5'-ATGGCGTCCAAGACTACCAC-3'
Rex-1 reverse	5'-ACTTTGCCCCCAAACTCTTT-3'

**Three germ layer genes**	
AFP forward	5'-AGCGGCTGACATTATTATCG-3'
AFP reverse	5'-GCAGGAGGGACATATGTTTC-3'
GATA4 forward	5'-GCCAGTCTACGTGCCCACA-3'
GATA4 reverse	5'-GGGTGTAAGCGGCTCCGTC-3'
GATA6 forward	5'-TCCACTCGTGTCTGCTTTTG-3'
GATA6 reverse	5'-CCCTTCCCTTCCATCTTCTC-3'
Sox17 forward	5'-CCTGGGTTTTTGTTGTTGCT-3'
Sox17 reverse	5'-GAGGAAGCTGTTTTGGGACA-3'
FOXA2 forward	5'-CTACGCCAACATGAACTCCA-3'
FOXA2 reverse	5'-CGGTAGAAGGGGAAGAGGTC-3'
Brachyury T (T) forward	5'-ACCCAGTTCATAGCGGTGAC-3'
Brachyury T (T) reverse	5'-ATGAGGATTTGCAGGTGGAC-3'
MIXL1 forward	5'-GGTACCCCGACATCCACTT-3'
MIXL1 reverse	5'-GCCTGTTCTGGAACCATACCT-3'
TBX1 forward	5'-CGCCGGTGAAGAAGAACG-3'
TBX1 reverse	5'-CACTTGGAAGGTGGGAAACA-3'
c-actin forward	5'-TGTGCTAGACAGGAACTCAGAT-3'
c-actin reverse	5'-GATGAGTCCAGAGTACTCCAAA-3'
Nestin forward	5'-AACAGCGACGGAGGTCTCTA-3'
Nestin reverse	5'-TTCTCTTGTCCCGCAGACTT-3'
Sox1 forward	5'-GGACTCTCTCTGAGGTTCTTTG-3'
Sox1 reverse	5'-GGCCCACATCCTAATCTTGA-3'
NeuroD1 forward	5'-ATCTTGCACAGGGAGTCACC-3'
NeuroD1 reverse	5'-TACTGCCGTCCAGTCCCATA-3'
Pax6 forward	5'-GCTCGGTGGTGTCTTTGTCA-3'
Pax6 reverse	5'-CAGAATTCGGGAAATGTCGC-3'
**Control**	
18S RNA forward	5'-CAGCCACCCGAGATTGAGCA-3'
18S RNA reverse	5'-TAGTAGCGACGGGCGGTGTG-3'
